# Acoustic Emission Signatures as Predictors of Laser Weld Quality: A Machine Learning Framework for Real-Time Laser Welding Quality Assessment

**DOI:** 10.3390/ma19194089

**Published:** 2026-09-24

**Authors:** Mohammadhossein Norouzian, Marko Orosnjak, Fadik Aslan, Ralph Useldinger, Slawomir Kedziora

**Affiliations:** 1 Department of Engineering, Faculty of Science, Technology and Medicine (FSTM), University of Luxembourg, 6, rue Coudenhove-Kalergi, L-1359 Luxembourg, Luxembourg; marko.orosnjak@uni.lu (M.O.); ralph.useldinger@uni.lu (R.U.); slawomir.kedziora@uni.lu (S.K.); 2 CERATIZIT Luxembourg Sarl, 101, rue de Holzem, L-8232 Luxembourg, Luxembourg

**Keywords:** laser welding, weld quality assessment, Machine Learning, time-frequency feature extraction, unsupervised clustering, Industry 4.0 manufacturing

## Abstract

Ensuring weld integrity in dissimilar-material joints remains a critical challenge, as current quality control relies heavily on destructive post-process inspection. Here, we assess airborne Acoustic Emission (AE) monitoring for in-process quality evaluation in laser welding of cemented carbide-steel. AE recorded over 11 welding conditions that were described by time- and frequency-domain features, partitioned by unsupervised clustering into three regimes, unstable weld (UW), quality weld (QW) and crack-defective weld (CDW), and classified by four shallow Machine Learning models. Because 10 ms windows from a single weld are not independent observations, performance is reported using grouped cross-validation, in which each window from a held-out weld is excluded from training, at two levels: leave-one-reading-out (LORO) and the stricter leave-one-condition-out (LOCO). Crack-defective welds caused by lateral beam offset were identified in all AE readings under both schemes (window-level AUC 0.987–1.000). Incomplete versus continuous bonding was separated with 0.846 reading-level accuracy under LOCO, and a combined two-stage model reached 0.857 accuracy and 0.883 balanced accuracy (permutation 0.002). Amplitude-domain features increased monotonically with heat input and saturated near 35 J/mm, so the acoustic signature varies gradually with heat input rather than forming a distinct class, whereas beam offset altered the spectral signature. Shear strength and fracture surface analyses corroborated the physical interpretation of the three regimes. The results establish that AE encodes an interpretable and predictive fingerprint of weld integrity and delimits the conditions under which it generalises to unseen welds.

## 1. Introduction

Cutting tools are foundational to modern manufacturing, determining the precision, productivity, and economic efficiency of operations [1,2]. Although they represent only a fraction of production costs, the reliability of these tools directly influences the throughput and quality of the entire manufacturing process. Among the diverse types of cutting tools, blades in oscillating multi-tools are widely used and require versatility and precision. These blades are typically subjected to high cyclic loading, making the durability of their design and material composition critical. The cutting blade used in this application is typically made of cemented carbide for the active cutting edge and steel for the supporting base. However, failures in these cutting tools often originate from imperfections at the cemented carbide-steel joint interface, which can lead to costly downtime, scrapped components, and safety risks for operators [3]. Hence, ensuring the integrity of dissimilar-material joints is not only an engineering challenge but also a pressing concern for industrial competitiveness and societal productivity [4].

Laser welding has emerged as a preferred joining technique for a variety of materials, offering narrow Heat Affected Zones, design flexibility, and compatibility with automated production [5]. Yet, the process is vulnerable to defects such as lack of fusion [6], porosity [7], and crack formation [8], which remain critical barriers. These defects can critically undermine performance but are occasionally undetectable through surface inspection. In laser welding of cemented carbide and steel, challenges mainly arise from their mismatch in thermal, mechanical and chemical properties, which promotes carbon diffusion and the formation of brittle phases in the weld zone [9,10]. Prior work highlights the critical influence of process parameters (e.g., laser power, beam velocity) [11]. Specifically, in butt welding, increasing spot size, optimising heat input, and avoiding direct laser beam exposure have been shown to improve bonding and mechanical strength while mitigating porosity and the formation of brittle intermetallic compounds at the weld interface [11,12,13]. These developments underscore the broader challenge of achieving reliable, defect-free welds when joining these materials.

The current quality assurance practices remain heavily reliant on post-weld inspections, which often involve destructive testing [14,15]. While effective for detecting major defects, such approaches are time- and resource-intensive and do not guarantee comprehensive quality verification across all manufactured parts [16]. The absence of reliable, real-time quality assessment limits the broader adoption of advanced welding processes in production [17,18,19]. In response, a wide range of sensor technologies has been explored: high-speed cameras [20,21,22], photodiodes [23,24], X-ray imaging [25,26,27], computer-vision methods [28,29,30], enabling detailed characterisation of weld pool dynamics. Recent advances extend these concepts to hybrid sensing, where multimodal data (e.g., synchrotron-based imaging) combined with Machine Learning (ML) models achieve >90% accuracy in predicting defects [31]. Analyses of AE (Acoustic Emission) have also provided validated frameworks for in-line monitoring in lithium-ion battery welding [32]. Moreover, multimodal fusion of AE and optical spectra has been shown to reduce noise sensitivity and improve precision in steel laser welding [33], while time-frequency analysis of AE confirmed that acoustic signatures encode distinct physical mechanisms of weld dynamics [34].

Indeed, AE sensing has gained increasing attention as a cost-effective and non-invasive alternative [32,35,36,37,38,39]. Primarily because AE signals capture transient elastic waves generated by keyhole fluctuations, offering a sensitive indicator of weld quality classification [37,40,41,42,43]. Many have demonstrated the feasibility of AE-based classification in welding of aluminium alloys, achieving ~87% accuracy with time-domain features [17]; validated in studies [41,43]. Mathivanan et al. [44] extended this approach to Cu-Al welds, achieving ~91% accuracy using neural networks. Lv et al. [45] developed an AE-based system for monitoring and controlling weld penetration, achieving 80–90% recognition accuracy with a BPANN (Backpropagation Artificial Neural Network). These contributions further reinforce the potential of AE not only for defect detection but also for closed-loop control in automated welding. Nevertheless, applications to cemented carbide-steel joints remain largely absent despite their industrial relevance. Here, we focus primarily on AE studies (Table 1), while detailed reviews of other monitoring practices are described elsewhere [46,47,48,49,50,51].

This study aims to investigate the laser welding of cemented carbide (WC-3 wt.% Co) to steel (0.4 wt.% C) and to develop a framework for weld quality prediction using AE signature-based features combined with ML. The proposed approach is intended to reduce reliance on post-production inspection by enabling in-process quality evaluation. AE monitoring captures the dynamic response of the weld zone, with signal features extracted from both the time and frequency domains. These features are first analysed using unsupervised methods to identify intrinsic acoustic regimes, and the resulting categories are then interpreted in light of metallographic and mechanical evidence. Because the AE dataset consists of short signal windows drawn from a limited set of physical welds, we emphasise the validation protocol: classifier performance is estimated using grouped cross-validation in which no window from a held-out weld contributes to training, so that the reported accuracy reflects prediction on unseen welds rather than reproduction of the clustering. By integrating experimental observations with intelligent monitoring, we establish a methodology that enhances weld quality evaluation and supports more efficient manufacturing practices. To our knowledge, no prior research has examined the use of both AE-based time- and frequency-domain features for quality assessment in laser welding using the proposed materials.

## 2. Materials and Methods

### 2.1. Data Framework Overview

The data analysis (Figure 1) consists of three parts: (1) Raw Dataset, (2) feature space, and (3) ML classifiers. The first part involves non-destructive data acquisition, signal (pre)processing, and feature extraction. Non-destructive data acquisition considers AE data from laser welding. The second part, feature space, utilises an unsupervised learning tool designed to reduce dimensionality and enhance the interpretability of a high-dimensional feature set. After obtaining a new feature set leveraging Principal Component Analysis (PCA), we proceed to identify clusters using Hierarchical Clustering on Principal Components (HCPC). These clusters are subsequently interpreted and assigned to weld-quality categories based on representative weld observations and metallographic evidence. The resulting labels are then used for training the classifiers. The third part centres on training shallow learning algorithms. Performance is assessed with standard metrics to enable robust comparisons. Hyperparameter tuning and architecture optimisation are conducted repeatedly, guided by validation results. Lastly, ablation studies and Genetic Algorithm (GA) optimisations are employed to refine the best classifier and clarify the significance of individual features.

### 2.2. Experimental Setup and Data Preprocessing

#### 2.2.1. Laser and Sensor Experimental Setup

A 2000 W continuous-wave Nd: YAG laser, Trumpf TrueDisk (Ditzingen, Germany), served as the laser source, delivering a constant average power during the laser welding process. The laser operates at a wavelength of 1030 nm with a beam quality of 2 mm/mrad. Laser energy is transmitted via an optical fibre with a 200 μm core diameter to a scanning head. With a focal length of 160 mm and a collimator length of 90 mm, the resulting laser spot diameter is approximately 356 μm.

Acoustic signals were acquired using a 1/2-inch prepolarised free-field microphone (Model 378B02, PCB Piezotronics, Depew, NY, USA) positioned near the welding zone (see Figure 2). Because the sensor measures airborne sound pressure, the term Acoustic Emission (AE) in this study refers specifically to transient airborne Acoustic Emissions generated during welding. The microphone has a frequency response of 3.75 Hz–20 kHz, a dynamic range of 15.5–146 dB SPL, and a sensitivity of 50 mV/Pa. The signals were acquired at a sampling rate of 51.2 kHz.

The laser welding process was performed on dissimilar materials: Cr-Mn steel (0.4 wt.% C) and cemented carbide consisting of tungsten carbide (WC) with 3 wt.% Co as the binder supplied by Ceratizit (Mamer, Luxembourg). The material properties are summarised in Table 2. Both specimens were clamped in a fixture to form a butt-weld joint. The steel sheet is 1.0 mm thick, whereas the cemented carbide component is 1.2 mm thick. To achieve precise alignment for butt welding, the fixture was designed with a height offset, placing the cemented carbide platform 0.2 mm lower than the steel. During the laser welding process, argon shielding gas at 10 L/min is supplied to the weld zone to protect the melt pool [20].

During the welding process, the laser beam trajectory was continuously controlled at a constant linear travel speed. Variations were introduced in key parameters, including laser power and beam position. The heat energy input was estimated based on the nominal laser power and welding speed. It is calculated using the following equation [53]:

(1) $$Q=\frac{P}{S},$$

where Q represents the heat input (J/mm), P denotes the laser power (W), and S is the welding speed (mm/s). Table 3 summarises the selected process parameters.

These conditions were defined by heat inputs ranging from 31.1 to 38.2 J/mm, with the laser beam intentionally shifted toward the cemented carbide side from the steel-cemented carbide interface by +0.25 and +0.5 mm to investigate the influence of beam position on weld quality. These offsets were selected based on preliminary welding trials and the reported sensitivity of this joint to the laser beam position relative to the interface [20]. Based on these variations, three main cases (A, B, and C) and 11 conditions were selected. These conditions were defined by heat inputs ranging from 31.1 to 38.2 J/mm, with the laser beam intentionally shifted toward the cemented carbide side from the steel-cemented carbide interface by +0.25 and +0.5 mm to investigate the influence of beam position on weld quality. Based on these variations, three main cases (A, B, and C) and 42 readings were selected.

#### 2.2.2. Acoustic Emission Preprocessing

For a detailed description of the AE signal preprocessing, we illustrate the steps in a flowchart (Figure 3). The data acquisition process began by initialising the laser welding procedure. The AE signals were recorded via the microphone; the raw data were extracted from the NI DAQExpress (v5.1) software and imported into Python 3.7.4. The code first extracts the time and amplitude data, calculates the sampling rate, and isolates the weld process, i.e., the Region of Interest (ROI) corresponding to the welding period. Time-domain and frequency-domain representations of the ROI were then generated to visualise signal characteristics within the 0–20 kHz range. These plots and the processed waveform were saved for further analysis. Following signal preprocessing, the AE data were segmented into consecutive 10 ms sequences to capture transient characteristics within the welding period. For each segment, 18 features were extracted from the signal to comprehensively characterise AE behaviour. Some data segments were excluded from the dataset due to missing or incomplete waveforms caused by acquisition dropouts. The 42 readings from 11 conditions were segmented into consecutive 10 ms AE windows. After excluding erroneous or incomplete segments, 1865 AE segments were retained, from which 33,570 valid data points were obtained for the time- and frequency-domain feature analysis. The description of each feature used for the analysis is explained in the following subsection.

#### 2.2.3. Feature Extraction

Time-domain features provide a straightforward approach to quantifying the temporal characteristics of the raw signal. Similar to the feature extraction used in prior studies [54,55,56,57], we summarise time-domain features alongside analytical descriptions that capture various aspects of signal magnitude, variability, and behaviour (Table 4). For instance, the Root Mean Square (RMS) amplitude indicates signal power, while the mean, variance, and standard deviation quantify central tendency and dispersion. Energy and mean energy are used to characterise the total and average signal strength, providing insight into the overall signal activity. The zero-crossing rate (ZCR) is the frequency of sign changes, indicating the signal’s oscillations. Lastly, short-term amplitude, peak-to-peak, min, and max values offer insight into local transients and signal extrema.

In contrast to time-domain features, which characterise the signal over time, frequency-domain features provide a complementary view of how energy is distributed across the spectrum. As detailed in Table 5, the features are computed via one-sided Fast Fourier Transform (FFT), excluding the DC bin. To describe the features, let *x*[*n*], *n* = 0, … *N* − 1, be sampled at *f_s_* [Hz]. Using the one-sided FFT *X*[*k*] = rFFT{*x*[*n*]} with frequency bins $f_{k}=\frac{k}{N}f_{s}$. The NumPy uses *k* = 0, …, [*N*/2]), thus for non-negative spectral weights ${\omega_{k}=\left|X[k]\right|}^{p}$ (magnitude *p* = 1, power *p* = 2). In this case, the features are computed from one-sided FFTs of the AE signal using power spectral weights (${\omega_{k}=\left|X[k]\right|}^{2}$).

The spectral centroid is used to measure the centre of mass of the frequency. As such, they reflect dominant frequency regions within the signal. Next, the spectral bandwidth describes the spread of spectral energy around the respective centroid, indicating variability. To quantify energy concentration, the spectral roll-off threshold α = 0.85 is defined and used to estimate the total spectral energy. The corresponding spectral roll-off is the frequency below which this percentage is accumulated. These features are used to distinguish between low- and high-frequency-dominant signals. Cumulative magnitude aggregates the total spectral amplitude, whereas RMS frequency provides a power-weighted average, analogous to RMS in the time domain. Together, these features capture global and local spectral energy concentrations, enabling detailed analysis of the AE welding signal.

### 2.3. Feature Space

To mitigate feature redundancy and facilitate downstream analysis, the high-dimensional time- and frequency-domain feature space was subjected to Principal Component Analysis (PCA). PCA is used to reduce a high-dimensional feature space into a lower-dimensional space of linearly uncorrelated variables, i.e., PCs (Principal Components). For the sake of consistency and explainability, let us consider the obtained feature space X ϵ ℝ^n×p^, where *n* is the number of observations and *p* is the number of features. Each feature is zero-centred and scaled to unit variance. The PCA transformation is performed through eigendecomposition of the covariance matrix:

(2) $$\Sigma =\frac{1}{n-1}X^{⊤}X.$$

The eigendecomposition yields:

(3) $$\Sigma =V\Lambda V^{⊤},$$

where **V** ϵ R^p×p^ is the matrix of eigenvectors (principal axes), **Λ** = diag(λ_1_, λ_2_, …, λ*_p_*) is the diagonal matrix of eigenvalues, with λ_1_ ≥ λ_2_ ≥ … ≥ λ*_p_* ≥ 0, and λ*_j_* represents the variance explained by the *j*-th component. The Principal Component **Z** ϵ R^n×p^ is obtained by projecting the original data onto the eigenvector basis:

(4) $$Z=XV\;,Z\in R^{n\times p}$$

such that the explained variance ratio for each component is represented by:

(5) $$\eta_{j}=\frac{\lambda_{j}}{\sum_{k=1}^{p}\lambda_{k}}.$$

To select the optimal number of components *m* < *p*, we retained those satisfying the condition:

(6) $$\sum_{j=1}^{m}\eta_{j}\geq \tau \;,$$

where *τ* is typically set at ≥70% in the literature. Here, we retain the components that explain at least 85% of the variance. This truncation yields the reduced matrix:

(7) $$Z_{m}=XV_{m}\;,\;Z_{m}\in R^{n\times m}$$

which is actually obtained as a lower-dimensional representation of the original feature space. The PCA was performed in RStudio (v.2025.05.1-513) in the FactoMineR package (v2.12) [58].

After plotting the data in PCA space, HCPC (Hierarchical Clustering on Principal Components) is applied as a hybrid method that combines dimensionality reduction, hierarchical clustering, and optional partition-based clustering. This ensures that clustering is performed on uncorrelated, variance-maximising features, thus improving the robustness and interpretability. The clustering is performed iteratively by merging clusters that yield the minimum increase in within-cluster variance, in accordance with Ward’s minimum variance criterion. For analytical description, we define *D* = {**z**_1_, **z**_2_, …, **z***_n_*} as a set of *n* observations in PCA space, where **z***_i_* ϵ R*^m^*, and *C* = {*C*_1_, *C*_2_, …, *C_K_*} is the current partition of clusters, ${\bar{z}}^{\left(k\right)}$ is the centroid of cluster *C*_k_. The within-cluster inertia *W* is then defined as:

(8) $$W=\sum_{k=1}^{K}\sum_{z_{i}\in C_{k}}{\left\|z_{i}-{\bar{z}}^{\left(k\right)}\right\|}^{2}.$$

At each agglomerative step of Ward’s minimum variance, the algorithm selects the pair of clusters (*C_r_*, *C_s_*) whose merging minimises the increase in within-cluster inertia:

(9) $$\Delta W_{r,s}=\frac{\left|C_{r}\right|\cdot \left|C_{s}\right|}{\left|C_{r}\right|+\left|C_{s}\right|}\cdot \;{\left\|{\bar{z}}^{\left(r\right)}-{\bar{z}}^{\left(s\right)}\right\|}^{2}.$$

Here, |*C_r_*| and |*C_s_*| are the sizes of clusters. The criterion ensures that merges are chosen to minimise the increase in variance within clusters, rather than being based solely on centroid distance. The process is repeated iteratively, yielding a dendrogram from *n* singleton clusters down to a single cluster. The optimal number of clusters is determined by examining the gain in between-cluster inertia at each cut. Total inertia is decomposed as *T* = *W* + *B*, where *B* is the between-cluster inertia. The relative inertia gain when reducing the partition from *K+1* to *K* clusters is:

(10) $$\Delta B\left(K\right)=\frac{B_{K}-B_{K+1}}{T}.$$

A sharp drop in Δ*B*(*K*) indicates that two well-separated clusters have been merged, thus providing a criterion for selecting the number of clusters. As the final step in obtaining a feature space with expert-based labelling, the clusters identified through unsupervised PCA-HCPC analysis were labelled by experts. The goal was to associate each cluster with a domain-relevant weld quality class. This was achieved using statistical diagnosis provided by the HCPC() function in FactoMineR (v2.1.7) [58,59]. The specificity analysis and paragon estimation guided the interpretation and validation of clusters. Integrating data-driven discovery with expert validation helped us ensure that the resulting classes are both statistically justified and operationally meaningful.

### 2.4. Machine Learning Classifiers

#### 2.4.1. Shallow Learning Classifiers

A range of shallow learning models was used, selected for their interpretability and established performance. Given the small sample size and computational complexity, it has been reported that shallow classifiers outperform deep learning models [60]. Here, we employed four shallow classifiers: Random Forest (RF), Support Vector Machine (SVM), Multinomial Logistic Regression (MNLR), and Artificial Neural Network (ANN). For an analytical description of all included classifiers, the reader is referred to prior studies [61,62]. However, for the sake of interpretability and reproducibility, we provide the parameters and hyperparameters used to model the classifiers.

RF is designed according to the following parameters: proportion of training data per tree (sampsize) is 50% of the training data per bootstrap sample; number of trees (ntree) is set to 100 (optimised maximum); number of variables tried at each split (mtry) is set to automatic for classification (default is square root of number of predictors). Gini impurity is used by default for classification. The minimum size of a terminal node is 1, while the maximum number of nodes is unspecified. Also, the maximum number of terminal nodes, class weights, and sampling stratification are not restricted. Out-of-bag (OOB) error estimation was automatically computed. The analysis relies on randomForest package [63].

Next, the SVM is built with the following parameters: the kernel is linear; the cost of constraint violation is 5; and the termination tolerance is 0.001. Other default settings in SVM classification are as follows: the shrinking heuristic is enabled, probability estimates are disabled, cross-validation is set to 0, class weights are equal, the maximum number of iterations is set to 1 × 10^5^, and predictors are scaled to have zero mean and unit variance. The package we used is e1071, which was used e1071 (v.1.7-16) based on libsvm [64].

The MNLR is performed via nnet (v7.3-20), considering the following parameters: the link function multinom() defaults to the generalised logit link for multinomial outcomes. The intercept is included. Other default settings of nnet::multinom() include iteratively reweighted least squares in the optimisation algorithm with Newton-Raphson steps; maximum iterations are set to 100 per optimisation run; relative tolerance 1 × 10^−8^; convergence tolerance 1 × 10^−4^ as absolute tolerance to convergence criterion for likelihood improvement; starting values for all coefficients are 0; penalty is not applied; hessian matrix is by default not computed (hess = false) [65].

The ANN is implemented as a Multi-Layer Perceptron (MLP). The neuralnet (v1.44.2) R package is used [66]. We start by using a standard ANN with BP (Backpropagation), but extend it with Rpror+ (Resilient BP with weight backtracking) [67], Rpror- (Resilient BP without weight backtracking) [68], Gprop-sag (Globally convergent algorithm with the smallest absolute gradient), and Gprop-slr (Globally convergent algorithm with the smallest learning rate) [69,70]. The following activation functions are tested: logistic sigmoid, ReLU, Leaky ReLU (LReLU), inverse tangent, and softmax. Learning rate is set to 0.1 (backprop); the threshold 1.0 for stopping criteria based on partial derivatives; maximum number of training repetitions is 1 × 10^4^ (stepmax). The network architecture search is performed with Genetic Algorithm (GA) with the following parameters: population size = 20; generations = 10; maximum number of hidden layers is set to 2 (to maintain shallow learning domain); maximum number of hidden nodes per layer = 10; parent selection = roulette wheel; crossover = uniform; mutation = reset with probability 10%; survival method is fitness-based with 10% elitism. Other default settings include randomly initialised weights drawn from a uniform distribution [−0.5, 0.5], bias nodes (included by default), and a stopping rule that terminates training when partial derivatives reach stepmax.

For all included classifiers, the features are scaled using *z*-score standardisation (mean 0, standard deviation 1). To remove the randomness and ensure repeatability, the seed is set to 1234. The data is split into training, validation, and test sets in a 60/20/20 split. Given the available information, and to ensure the reproducibility of results, all included parameters can be set manually using the open-source software JASP (v.0.19.3), with no coding required.

#### 2.4.2. Evaluation Metrics

To assess classification performance in the three-class setting, we distinguished between the classifiers’ internal multiclass learning strategies and the class-wise evaluation procedure. The classifiers retained their native multiclass implementations rather than being uniformly reformulated as one-vs-rest models. In particular, the SVM implementation uses a one-vs-one decomposition for multiclass learning, whereas RF, Multinomial Logistic Regression, and ANN directly accommodate multiclass outcomes. For class-specific evaluation, a one-vs-rest formulation was used: each weld-quality class (UW, QW, and CDW) was treated in turn as the positive class, while the remaining two classes were jointly treated as the negative class. Precision, recall, and F1-score were calculated accordingly. Hence, the following list of evaluation metrics is used:

(11) $$Accuracy=\frac{TP+TN}{TP+TN+FP+FN},$$

where *TP* is True Positive predicted outcomes, *TN* is True Negative, *FP* is False Positive, and *FN* is False Negative predicted outcomes. Note that the evaluation of accuracy is performed on a specific class-type label *c*. Next, the precision:

(12) $$Precision=\frac{TP}{TP+FP},$$

The recall, i.e., sensitivity, is estimated:

(13) $$Recall=\frac{TP}{TP+FN},$$

And F1-score:

(14) $$F1\;score=2\frac{Precision\times Recall}{Precision+Recall},$$

were used as evaluation metrics for the models. In addition, given the range of available metrics and considering class imbalances, we used the Area Under the Receiver Operating Characteristic Curve (AUC-ROC), commonly referred to as the AUC metric, on a one-vs-rest evaluation basis, as we have a multiclass problem. To evaluate feature importance, the MDL (Mean Dropout Loss) is used, which is based on the mean cross-entropy loss between predicted class probabilities and true labels. Hence, the metric is used to evaluate the contribution and importance of individual features per label. Lastly, for the ablation study, Recursive Feature Elimination (RFE) is used to select the classifier with the best performance and the fewest features. In addition, we tested ANN-MLP with different optimisation (hyper)parameters and discussed the findings.

#### 2.4.3. Validation Design and Units of Independence

The dataset comprises 1865 10 ms windows extracted from 42 AE readings recorded across 11 welding conditions (Table 3). Windows from a single weld share process parameters, specimen, and acoustic path and are not independent observations, so a random split of windows places windows from the same weld on both sides of the split. Such a split measures how consistently a model reproduces the labelling within the observed welds; it is reported in Section 3.3 for comparability but is not a generalisation estimate.

Section 3.5 therefore uses grouped cross-validation at two levels of independence. Leave-one-reading-out (LORO) holds out one complete AE reading while other readings of the same parameter setting remain in training, estimating performance for a previously characterised process setting. Leave-one-condition-out (LOCO) holds out all readings of a welding condition, so no window recorded at that heat input or beam offset enters training; this estimates performance on an unseen process setting and is the stricter scheme. Standardisation is applied within training folds only, and the targets are the metallographic categories from Section 3.1, not the cluster memberships. Two decision units are distinguished: the individual window and the complete AE reading, for which a verdict is obtained by majority vote over its windows or from a model trained on features aggregated over the reading (the mean and standard deviation of each descriptor, 36 features for each of the 42 readings). Since the classes are imbalanced, we report balanced accuracy and macro-averaged F1 alongside accuracy and train class-balanced models; the majority-class reference at the reading level is 0.571. Significance is assessed by permuting labels between welding conditions and repeating the complete LOCO procedure 500 times, and confidence intervals by bootstrap resampling of readings (2000 replicates).

## 3. Results

### 3.1. Experimental Observation and Welding Analysis

The welds undergo visual inspection from the top and bottom views using a FUJIFILM X-PRO2 camera (Fujifilm Corporation, Tokyo, Japan). This helps identify surface irregularities and visible defects. Additionally, to gain a deeper understanding of the internal weld quality, a LEICA MEF 4M optical microscope (Leica Microsystems GmbH, Wetzlar, Germany) is used for the cross-section microstructural analysis. For this process, the welds are sectioned, mounted, and prepared using standard metallographic techniques, including grinding, polishing, and etching, before undergoing microscopic examination.

Figure 4 shows representative images of the top and bottom surfaces and cross-sectional microstructures for the different cases at the adopted magnifications. Case A refers to welds made with the laser spot aligned along the steel edge and a heat input in the range of 31.11–35.56 J/mm. In the top surface image, no obvious defects are visible, suggesting a relatively uniform weld bead. However, the bottom surface reveals a gap between the cemented carbide and steel, indicating insufficient bonding across the interface.

In case A, the cross-sectional image provides additional insights into this behaviour. While bonding appears near the top of the joint, the middle and lower parts of the interface show clear signs of poor bonding. Additionally, a void is visible within the weld cross-section, indicating a significant defect that could weaken the joint’s strength and reliability.

These features suggest that the weld pool experienced repeated keyhole formations, along with irregular oscillations within the molten region. The weak bonding observed in the A series is mainly due to the relatively low heat input, which was insufficient to ensure proper penetration of the laser beam across the steel-cemented carbide interface.

Increasing the heat input to the range of 36.44–38.22 J/mm (case B) significantly improved weld quality compared to case A. The front, bottom surfaces, and the cross-section in case B show a continuous bonding interface between the steel and the cemented carbide. Although a dendritic microstructure (resembling black tree-like formations) is observed at the interface following Murakami etching (prepared in the laboratory), indicative of cemented carbide elements’ diffusion into the weld zone and η-phase formation, no cracks, voids, or other discernible defects were detected, demonstrating that the welding process remained stable and robust (Figure 4-Case B). The combination of sufficient thermal energy and correct laser beam alignment ensured adequate penetration and diffusion at the interface, resulting in consistent metallurgical bonding.

In Case C, the heat input is maintained at the optimised value of 37.33 J/mm, while the laser beam is shifted toward the cemented carbide, overlapping it by 0.25 mm. Both top-view and cross-sectional images reveal crack propagation and porosity, which severely compromise the joint’s mechanical performance. The interaction of the laser beam with the cemented carbide promotes the diffusion of carbon and tungsten into the fusion zone, leading to the formation of intermetallic compounds and creating a brittle region within the weld. Evaluating insights from destructive methods and visual inspection, the microstructure was first analysed, and defects were identified. This is followed by an investigation of the underlying phenomena using unsupervised analysis of AE signals.

### 3.2. Unsupervised Modelling and Clustering

Dimensionality reduction and clustering are summarised in Figure 5. The first two Principal Components explain 87.51% of the variance of the 18-dimensional feature set (PC1 64.92%, PC2 22.59%; Figure 5B) and were retained for clustering. In the biplot (Figure 5A) the samples spread mainly along PC1, whose loadings are carried by the amplitude and energy descriptors (RMS, energy, amplitude standard deviation, peak-to-peak), whereas PC2 contrasts spectral localisation with spread (roll-off and centroid against bandwidth). The frequency-domain results show a shift from 2.2 to 2.4 kHz in CDW to approximately 3 kHz in UW and QW. Such low-kHz variations may reflect changes in keyhole and melt-pool dynamics and in laser-material interaction with steel, cemented carbide or both; without synchronised high-speed imaging, however, they are treated as discriminative spectral signatures rather than assigned to specific mechanisms.

HCPC on these components reduces noise and multicollinearity and stabilises Ward’s criterion. The gain in between-cluster inertia (Figure 5C) supports a three-cluster partition, with some overlap between two of the clusters along PC1 (Figure 5A). Clusters were assigned to the weld-quality categories using the HCPC diagnostics paragons and specificity: paragons are the samples closest to each cluster centroid in PC space, and specificity quantifies how unambiguously they belong to that cluster. Paragon IDs were traced back to the raw signals and the physical and metallographic evidence, and borderline samples were excluded from labelling due to low specificity. The cluster isolated along PC2, with the highest specificity and the 2.2–2.4 kHz spectral shift, was labelled CDW; of the two clusters separating along PC1, the one with higher-energy paragons was labelled QW, and the one with lower, more irregular energy was labelled UW.

Because this assignment maps an unsupervised partition onto physical categories, we quantified the agreement directly, using the metallographic outcome of each condition as an independent reference (Figure 5D, Table A1). Agreement is substantial but incomplete: purity 0.846 and adjusted Rand index 0.543. The CDW cluster contains all 210 windows recorded from the two beam-offset conditions and only 33 from the others, so it essentially recovers the crack-defective condition exactly. The boundary between the two remaining clusters, however, does not coincide with the metallographic bonding threshold: the QW cluster accounts for 78–94% of windows from B1-B3 but also 48% and 35% of those from A5 and A6 (34.67 and 35.56 J/mm), which metallography classifies as incompletely bonded, while A1–A4 remain 78–89% in the UW cluster. The partition, therefore, reflects a genuine acoustic structure that is only partly aligned with weld quality, a discrepancy traced in Section 4.3 to the gradual dependence of AE amplitude on heat input. It also follows that accuracies obtained against cluster labels quantify the reproducibility of the partition rather than the prediction of weld quality.

### 3.3. Reproducibility of the AE-Derived Partition (Window-Level Split)

We first quantify how consistently the four classifiers reproduce the AE-derived partition within the observed welds. The classifiers are trained on the feature set with labels derived from PCA-HCPC and expert labelling, using a 60/20/20 split at the 10 ms window level. The resulting properties are: RF (50 trees, mtry = 4), Multinomial Logistic Regression (logit link), SVM (cost = 1.88; 54 support vectors) and ANN-MLP (two hidden layers with nine and six nodes). All models reproduce the partition closely: RF (Accuracy_test_ = 0.981, OOB = 0.981 in Table 6), Multinomial Logistic Regression (Accuracy_test_ = 0.981, in Table 7), SVM (Accuracy_validation_ = 1.00, Accuracy_test_ = 0.984, in Table 8), and ANN-MLP (Accuracy_validation_ = 0.987, Accuracy_test_ = 0.987, in Table 9). The narrow results suggest that the three AE regimes are highly separable in the chosen feature space.

These values require careful interpretation. Because the split is applied to 10 ms windows rather than to welds, windows from the same weld appear in both the training and test partitions, and because the targets are cluster memberships rather than an independent physical measurement, the quantity estimated is the internal consistency of the partition, not the accuracy with which AE predicts the quality of an unseen weld. Table 6, Table 7, Table 8 and Table 9 should therefore not be read as generalisation performance; the corresponding estimates are given in Section 3.5.

### 3.4. ANN-MLP Performance

The ANN-MLP reproduces the partition stably in validation and test (Figure 6A,B). Recursive Feature Elimination (RFE) shows that accuracy remains above 97% when the feature space is reduced from 18 to 10 features and drops markedly below nine (Figure 6C), with importance measured as Mean Dropout Loss over 50 permutations. Spectral roll-off, RMS, amplitude standard deviation, and the maximum and minimum amplitude consistently rank highest (Figure 6E), the frequency-domain feature contributing most to cluster separation. Network stability and feature reduction were further examined by Genetic Algorithm (GA) optimisation (Section 4.1).

### 3.5. Weld-Level Performance Under Grouped Cross-Validation

We now estimate how well AE predicts the independently determined quality of a weld that contributed no data to training. Because the two variables manipulated in the experiment are distinct, beam position affecting crack formation and heat input affecting bonding continuity, the task is treated in two stages: detection of the crack-defective condition and discrimination between incomplete and continuous bonding among the on-axis welds. Table 10 summarises the results.

The stricter protocol does not affect detection of the CDW condition. All 42 readings are classified correctly by Random Forest, SVM and Multinomial Logistic Regression under both schemes, with window-level accuracies of 0.969–0.995 and areas under the ROC curve of 0.987–1.000 (Figure 7). This holds when conditions C1 and C2 are withheld entirely, so the result does not depend on the model having seen the specific weld. Since only two beam-offset conditions were produced, however, the estimate rests on a narrow base and requires replication across further offset levels.

Discrimination between UW and CW is also well above chance but less complete: 33 of 39 on-axis readings are classified correctly under LOCO (0.846 accuracy, 0.825 balanced accuracy), with window-level accuracy of 0.837 and AUC of 0.913. Combining both stages yields 36 of 42 readings correct (0.857 accuracy, 0.883 balanced accuracy) under LOCO, with a window-level AUC of 0.943, rising to 0.954 under LORO. A model trained directly on the 36 aggregated reading-level features achieves 0.786 accuracy and 0.842 balanced accuracy under LOCO (bootstrap 95% CI: 0.643–0.905; majority-class reference: 0.571). Permuting the class labels between welding conditions and repeating the complete LOCO procedure 500 times yields a null distribution with a mean accuracy of 0.337 and a 95th percentile of 0.619 (balanced accuracy: 0.326 and 0.574), so both observed values correspond to *p* = 0.002, the smallest attainable value. The weld-level result is therefore not an artefact of the small number of groups.

The errors are concentrated in two respects (Table 11). First, they depend strongly on the length of the AE reading: all 27 readings comprising at least 25 windows (0.25 s) are classified correctly, whereas nine of the 15 shorter readings are not (Fisher’s exact test, *p* = 1.1 × 10^−5^). The effect is visible within conditions as well as between them; in B1, B2, B3, and A3, the misclassified readings are, in every case, the shortest ones, while readings from the 60–115 windows in the same conditions are correct. Second, the residual errors cluster around the bonding threshold: conditions A5 and A6 (34.67 and 35.56 J/mm), for which only short readings are available, account for four of the nine errors, and readings from A5-A6 are predicted as continuously bonded, while two short readings from B1 are predicted as incompletely bonded. The classification boundary learned from AE therefore lies near 35 J/mm rather than at the metallographic threshold near 36 J/mm, as examined in Section 4.3.

The effect is visible within conditions as well as between them; in B1, B2, B3, and A3, the misclassified readings are, in every case, the shortest ones, while readings from the 60–115 windows in the same conditions are correct. Second, the residual errors cluster around the bonding threshold: conditions A5 and A6 (34.67 and 35.56 J/mm), for which only short readings are available, account for four of the nine errors, and readings from A5–A6 are predicted as continuously bonded, while two short readings from B1 are predicted as incompletely bonded. The classification boundary learned from AE therefore lies near 35 J/mm rather than at the metallographic threshold near 36 J/mm, as examined in Section 4.3.

## 4. Discussion

### 4.1. Ablation Study and ANN-MLP Topology

The ANN-MLP results are reported in Figure 8. Four factors of the ANN-MLP were ablated on the window-level split: activation function, training algorithm, depth (one versus two hidden layers) and width. Activation-dominated: the smooth functions logistic-sigmoid and tanh gave the highest accuracies, softplus was intermediate, and ReLU, GeLU and Mish trailed (Appendix B, Table A2). Depth and width had a modest but consistent effect with the smooth functions, a second layer adding a small gain in generalisation. Among trainers, Rprop+ and the globally convergent Gprop-sag/slr variants were reliable across activations, with Gprop-slr marginally best, whereas plain Backpropagation required careful pairing and failed with tanh.

A Genetic Algorithm search over topology (population 20, 10 generations, at most two layers of at most ten nodes) with different parent-selection, crossover, mutation and survival operators favoured small, asymmetric two-layer layouts (e.g., 9;4, 4;9, 10;4) that matched or slightly exceeded the manual designs, with Validation Accuracy 0.990–1.000 and F1 0.984–0.992 across Rprop and Gprop trainers (Appendix B, Table A3). Multiple near-equivalent optima were found, and repeating the search with a second seed changed the discovered topologies but not the attainable Test Accuracy, which clustered in 0.984–0.992. The final classifier, two hidden layers of five and five nodes with logistic activation and Gprop-slr (Figure 8C), was selected on Validation Accuracy and maintained 0.98–0.99 Test Accuracy with a mean score of about 0.98 across accuracy, precision, recall, F1 and AUC (Figure 8A). Performance in this range was driven more by the feature subset than by the activation function.

The GA-selected features (Figure 8B) are consistent with earlier reports: amplitude standard deviation and peak-to-peak were most influential, followed by mean energy and spectral roll-off. Amplitude variability and extremes capture impulsive bursts, presumably from spatter, pore nucleation and keyhole instability; roll-off measures the proportion of high-frequency content; RMS and energy quantify emission intensity; bandwidth reflects process modulation. The t-SNE maps of the hidden layers (Figure 8D) show that the first layer preserves elongated, partly overlapping manifolds, while the second compresses them into compact regions, with residual overlap between UW and QW. A nine-feature model balances parsimony and accuracy, with performance plateauing between nine and 13 features in RFE.

### 4.2. Mechanical Strength of Predicted Welded Joints

The mechanical shear strength test was conducted to verify the weld quality classification. Using the SHIMADZU-SFL 50KN universal machine (Shimadzu Corporation, Kyoto, Japan), the maximum load before failure provided a direct measure of joint strength. In this setup, the steel body and weld zone were clamped between the upper and lower fixing plates, while a shear force was applied to the entire cemented carbide strip by a blade moving at 10 mm/min. Figure 9A illustrates the results of shear tests conducted on five specimens from each weld class, showing the variation in shear force.

Welch’s test revealed significant differences between classes: QW exhibited the highest shear strength (μ_QW_ ± SE = 2787.851 ± 226.03), outperforming both UW (t(21.543) = 5.125, *p* < 0.01, Cohen’s *d* = 1.934) and CDW (t(10.864) = −10.626, Cohen’s *d* = −4.585). Likewise, UW also differed significantly from CDW (t(20.243) = 5.358, *p* < 0.01, Cohen’s *d* = −1.784).

Assumptions were reasonably met: the Shapiro–Wilk test suggested no substantial deviation from normality (W = 0.892, *p* = 0.059), which was also supported by the QQ plot of residuals, while the Brown–Forsythe test confirmed homogeneity of variances (F(1, 14) = 3.034). The Shapiro–Wilk test suggests no violation; in the UW-CW case, the Brown–Forsythe test showed unequal variances (F(1, 22) = 6.098), justifying the use of Welch’s correction (Figure 9C).

Fractography analysis (Figure 9B) further corroborates these findings by showing distinct weld fracture morphologies across classes. QW samples exhibited a continuous interface with a thin layer of residual cemented carbide on the steel fracture surface, consistent with their markedly higher shear strength. In contrast, UW samples revealed incomplete fusion and a discontinuous thin layer of cemented carbide on the steel, and the CDW sample revealed that failure occurs in both the weld zone and the cemented carbide, characterised by relatively large residual cemented carbide particles and a brittle cleavage fracture within the weld zone. Together, the fractography analysis and statistical evidence converge to validate the weld quality classification, establishing QW as mechanically and structurally distinct from UW and CDW.

Figure 9D maps AE features to the cluster structure that underpins the weld classes. Frequency-domain descriptors spectral bandwidth, spectral roll-off, and RMS frequency most strongly discriminate Cluster 1 (CDW) from Clusters 2–3 (UW, QW), consistent with an off-axis laser beam shift (0.25–0.5 mm toward the cemented-carbide side) that excites microcrack events and shifts energy to higher frequencies (broader bandwidth, higher roll-off). In contrast, time-domain metrics RMS (amplitude), peak-to-peak, mean energy, amplitude deviation, and variance separate Cluster 3 (QW) from Clusters 1–2 when the beam remains on the steel side: stable keyhole dynamics and localised spatter generate sustained, high-energy transients, yielding elevated RMS and related amplitude-energy envelopes. Similarly, Cluster 2 (UW) shows that time-domain features dominate; however, cumulative magnitude (*t* = 52.774) emerges as the single most decisive feature (Cohen’s *d* = 2.489), reflecting markedly lower output of UW events compared with the sustained, high-energy activity of stable welds. Mechanistically, CDW reflects crack-dominated, spectrally “hard” bursts with comparatively lower integrated time-domain energy; UW reflects weak coupling and incomplete fusion with low amplitude and narrow spectra; QW exhibits energetically richer but spectrally more “balanced” emissions aligned with deeper penetration.

### 4.3. Heat Input and the Role of Observation Length

The contrast between the two classification tasks has a physical basis that also explains why the AE-derived partition does not align exactly with the metallographic bonding threshold. Across the nine on-axis conditions, amplitude-domain descriptors increase monotonically with heat input: Spearman correlations between reading-mean features and heat input (n = 39 readings) are 0.761 for log energy, 0.757 for short-term amplitude, 0.752 for energy, RMS and amplitude standard deviation, and −0.742 for minimum amplitude (all *p* < 0.001; Figure A1). Spectral descriptors correlate more weakly and in the opposite direction (zero-crossing rate −0.570, spectral roll-off −0.447, centroid −0.395, RMS frequency −0.368), while spectral bandwidth shows no association (rho = 0.000). Heat input is thus encoded principally in the amplitude domain, whereas beam offset, which is independent of heat input, is expressed spectrally.

The dependence saturates. Predicting heat input from AE by Random Forest regression under leave-one-condition-out validation yields a significant monotone association (Spearman rho = 0.817, *p* = 0.007 at condition level; rho = 0.640, *p* < 0.001 across readings), but predictions compress into 33.1–35.7 J/mm: they rise from A3 to A5 and then flatten, with B1–B3 predicted at 35.5–35.7 J/mm (Figure 10A,B). The share of windows predicted as continuously bonded follows the same shape, increasing from 0.09 to 0.18 across A1–A4 to 0.44 at A5 and 0.68–0.83 across B1-B3, without a discontinuity at the metallographic threshold (Figure 10C). Below saturation, additional heat input produces a more energetic and more variable acoustic signature as the melt pool grows; once bonding is continuous, further heat input changes the emission little. The transition from incomplete to continuous bonding consequently corresponds to a threshold in a gradually varying acoustic response rather than to a boundary between acoustically distinct regimes, which is why conditions A5, A6, and B1 are the hardest to separate and why the learned boundary lies near 35 J/mm. Crack formation from lateral beam offset is categorically different: it changes which material the beam principally couples into, and hence the spectral content, producing the clean separation of Section 3.5.

A second, practical determinant is the length of the observation. Every reading of at least 25 windows (0.25 s) was classified correctly, whereas nine of 15 shorter readings were not, and within individual conditions the misclassified readings were consistently the shortest. Aggregated features estimated from very few windows are noisy, and dispersion measures particularly so; the standard deviation of log energy is among the twelve most important reading-level features, behind the mean amplitude standard deviation and mean maximum amplitude. For deployment, this indicates a minimum acquisition length of roughly 0.25 s per verdict rather than a per-window decision, and it suggests that the residual errors reflect estimation noise as much as physical ambiguity, although reading length and welding condition are partly confounded here because A5 and A6 contributed only short readings.

## 5. Conclusions

This study presents and validates a data-driven framework for in-process quality assessment in laser welding of cemented carbide-steel joints. By combining Acoustic Emission monitoring with unsupervised clustering, feature-space optimisation, and shallow classifiers, and by evaluating the classifiers using grouped cross-validation in which no signal window from a held-out weld contributes to training, we determine which aspects of weld quality AE predicts on unseen welds. Crack-defective welds produced by lateral beam offset are identified in all 42 AE readings under both the leave-one-reading-out and the stricter leave-one-condition-out validation. Incomplete and continuous bonding are separated in 33 of 39 on-axis readings, and a combined two-stage model correctly classifies 36 of 42 readings (balanced accuracy = 0.883, permutation *p* = 0.002).

The discriminative features map onto the physics of weld formation. Amplitude-domain descriptors increase monotonically with heat input and saturate near 35 J/mm, so the boundary between incomplete and continuous bonding corresponds to a threshold on a gradual acoustic trend, and the residual errors concentrate around it; lateral beam offset alters the spectral signature and produces an acoustically distinct regime. Classification also requires a sufficient observation window, with every reading of at least 0.25 s classified correctly. Independent metallographic examination, shear-strength testing, and fracture-surface analysis support the physical interpretation of the three regimes: QW is associated with continuous bonding and the highest shear strength, while UW and CDW are associated with incomplete bonding and crack-related failure, respectively.

Taken together, the results show that Acoustic Emission provides an interpretable fingerprint of welding quality and delimits how far it extends: detection of crack-defective welds is supported as an in-process, non-destructive check, subject to replication across a wider range of beam offsets, whereas assessment of bonding quality is best treated as a graded estimate within a characterised parameter window. We further propose grouped cross-validation, with the weld as the unit of independence and the metallographic outcome as the target, as a minimum reporting standard for AE-based weld monitoring, since window-level splits substantially overstate performance on unseen welds.

### 5.1. Implications and Limitations

The findings of this study extend beyond the specific case of cemented carbide-steel laser welding. Scientifically, they demonstrate that Acoustic Emissions encode a mechanistic fingerprint of process quality, enabling direct links between transient signal features, weld microstructure, and mechanical performance. This provides a generalizable paradigm for decoding complex manufacturing processes, enabling in situ monitoring of hidden dynamics through physics-informed signal analysis.

Nevertheless, several limitations should be acknowledged. First, the experiments were conducted under controlled laboratory conditions, which may not capture the variability of industrial environments, including background noise, structural vibrations and thermomechanical transients from adjacent processes. Second, the WC-3 wt.% Co grade and the Cr-Mn steel grade were used within a narrow processing window. Third, and most consequentially, the dataset comprises 11 independent welding conditions and 42 AE readings; the 1865 windows are repeated observations of these welds, not independent weld observations. The crack-defective class derives from only two beam-offset conditions, so although it is detected in both validation schemes, the estimate rests on a narrow base. Conditions A5 and A6 contributed only two and three short readings, respectively, which limits precision at the bonding boundary, and reading length is therefore partly confounded with the welding condition. Fourth, the acoustic boundary between incomplete and continuous bonding lies near 35 J/mm rather than at the metallographic threshold near 36 J/mm, so conditions within this band are not reliably separable. Fifth, reliable verdicts require at least about 0.25 s of signal, making the method an end-of-pass rather than a per-window decision. Sixth, the three-category scheme may not capture finer gradations of defect morphology. Finally, robustness under domain shift, including new machines, fixtures and acoustic environments, remains to be established, and causality between specific AE signatures and defect modes warrants direct investigation, for example, by synchronised high-speed imaging.

### 5.2. Future Research

In the following studies, we consider expanding the framework to a broader range of welding parameters and cemented carbide grades, increasing the dataset and adding further sensing modalities such as thermal and high-speed imaging. Three priorities follow from the present results: increasing the number of independent welds, particularly additional beam-offset levels, so that crack detection is validated on a broader base; sampling heat input more finely within the 35–37 J/mm band, where the acoustic response saturates and the errors concentrate, with acquisition lengths sufficient for stable reading-level features; and treating heat input as a continuous regression target rather than bonding quality as a discrete class, which is more consistent with the observed physics. Also, given that our shear strength test was an a posteriori analysis, we consider a study predicting weld quality in terms of remaining useful life from AE signatures, bypassing unsupervised class assignment and categorising product quality from mechanical strength and life-cycle performance.

## Figures and Tables

**Figure 1:**
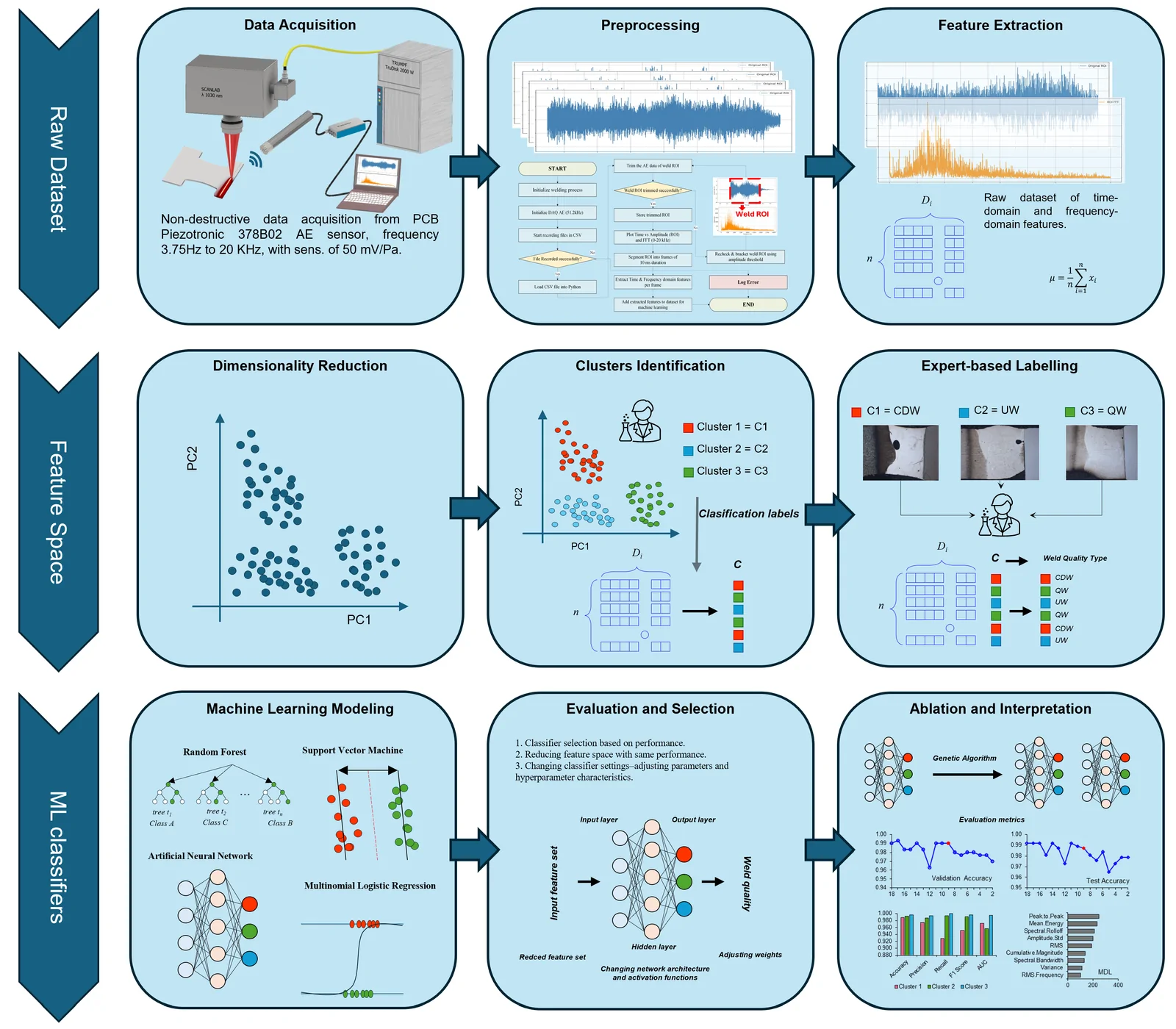
Illustrative overview of study design and data analysis framework.

**Figure 2:**
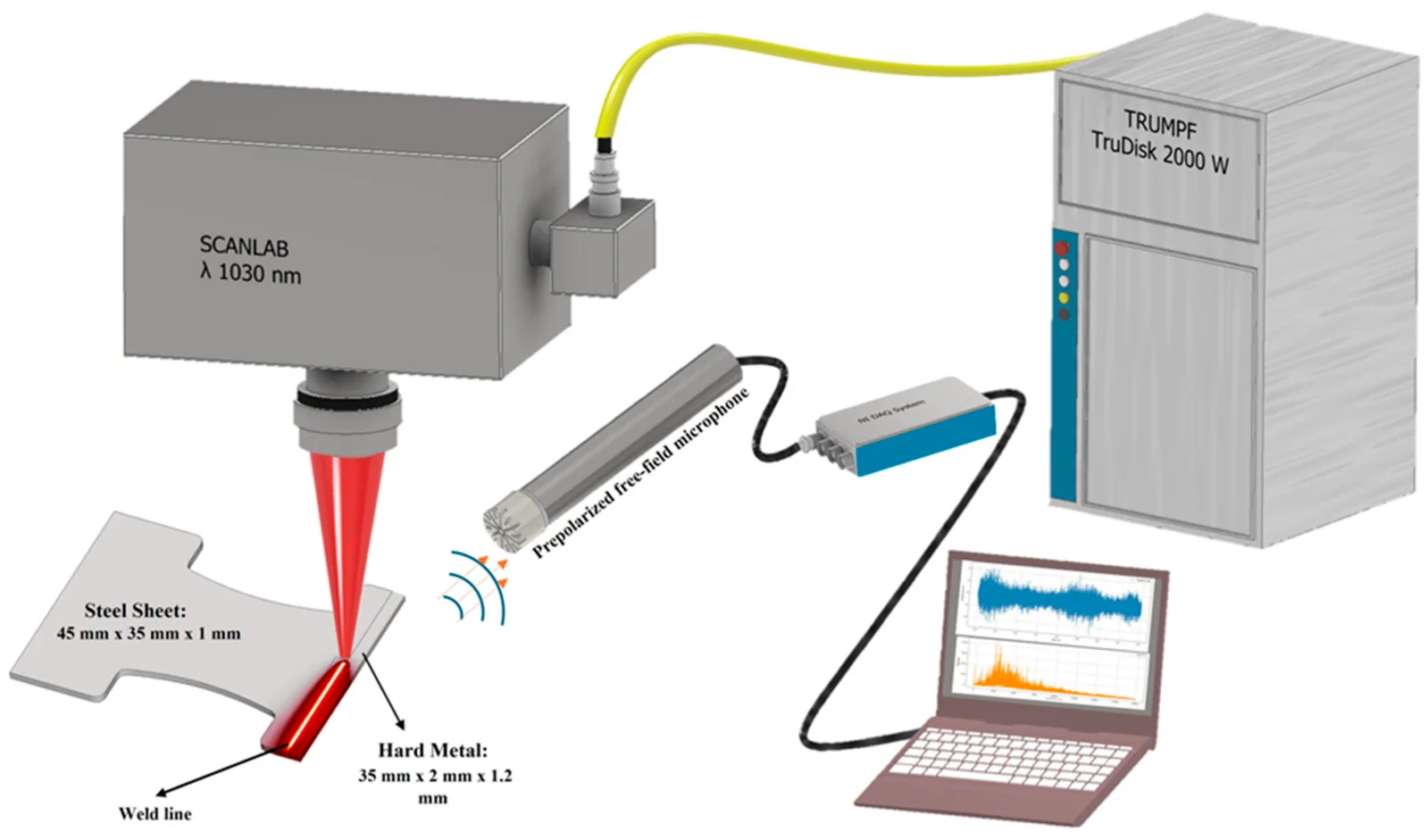
Experimental setup.

**Figure 3:**
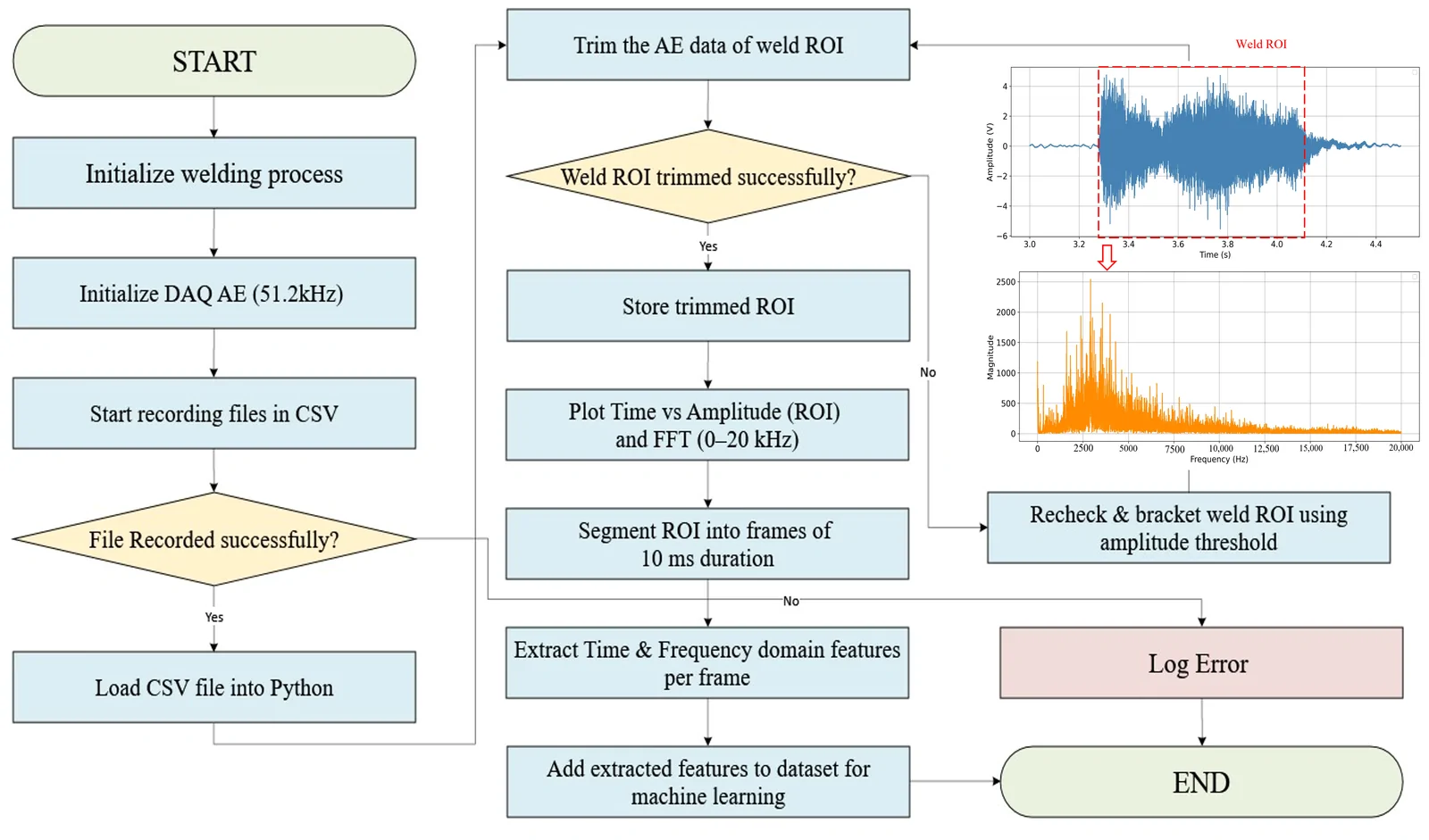
Data preprocessing.

**Figure 4:**
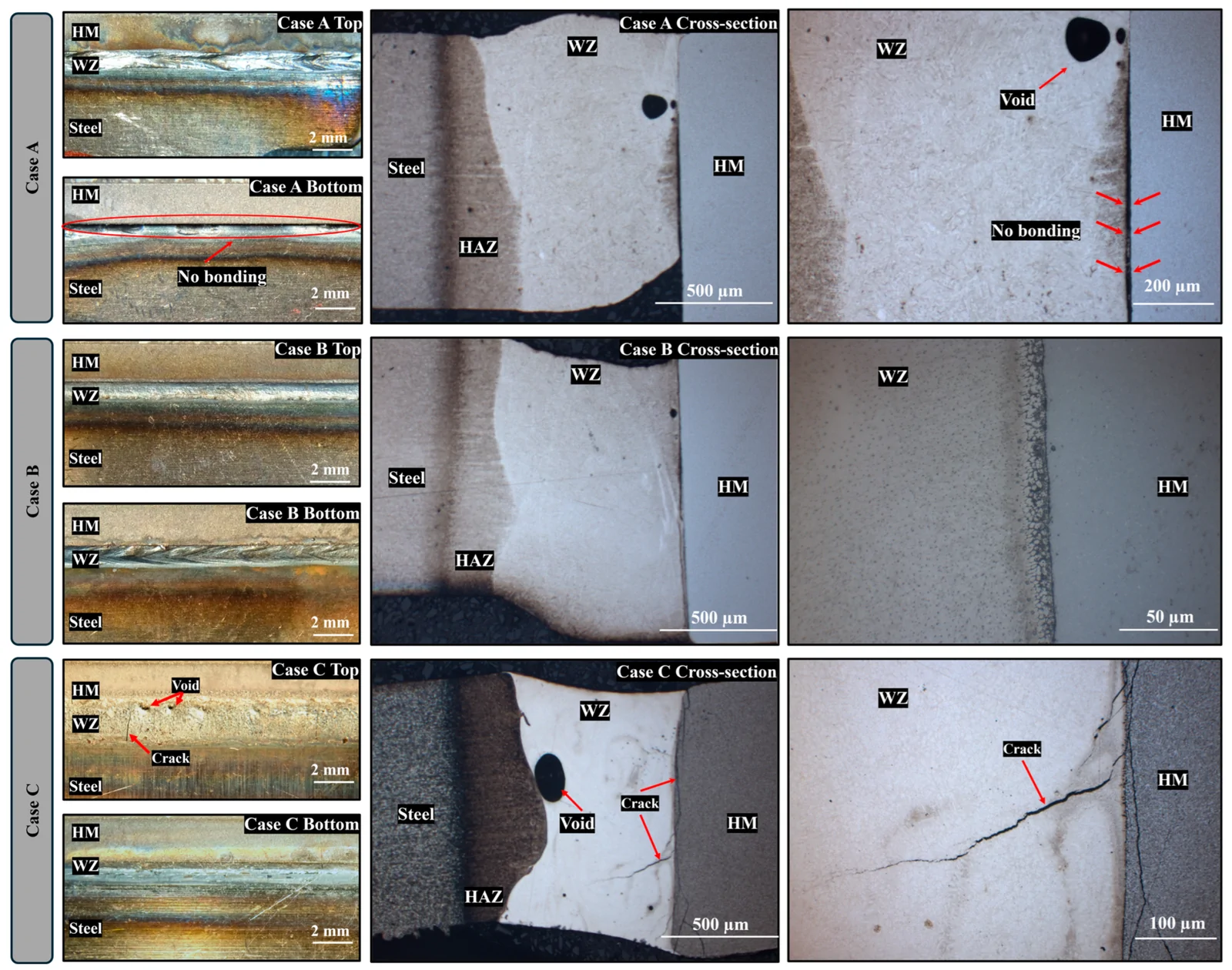
Visualisation of laser weld zones of cases A, B, and C. FUJIFILM X-PRO2 camera images of the weld joint, top and bottom, respectively (**left**). Cross-sections are analysed using the LEICA MEF 4 M light optical microscope for each case (**middle**) and at higher magnification (**right**). (Note: WZ = weld zone; HM = Hard Metal (Cemented Carbide); HAZ = Heat Affected Zone).

**Figure 5:**
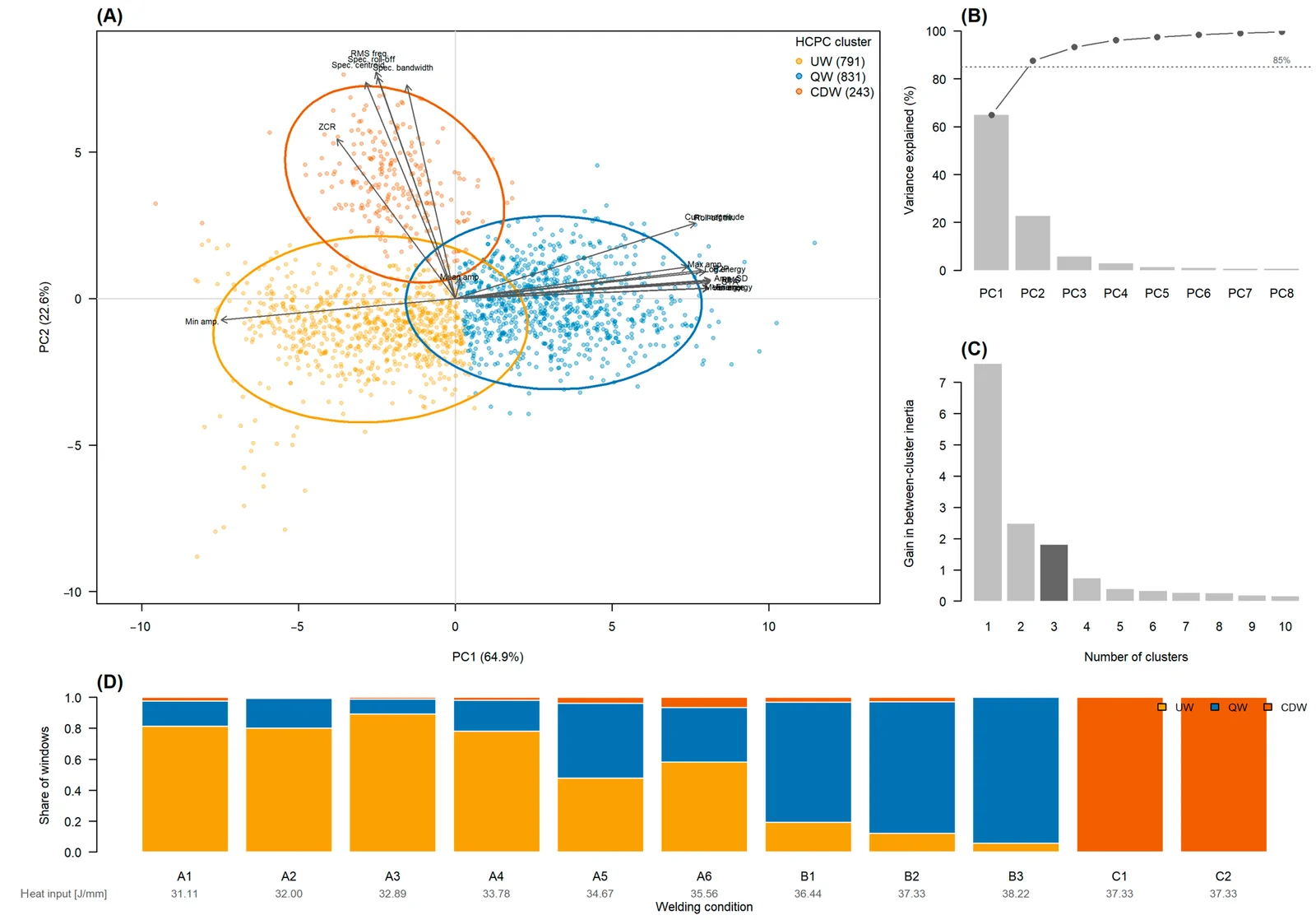
Principal Component Analysis and hierarchical clustering of the 1865 AE windows. (**A**) Biplot of PC1 (64.92%) and PC2 (22.59%) with HCPC clusters, 95% concentration ellipses and feature loadings; (**B**) variance explained by each component; (**C**) gain in between-cluster inertia used to select three clusters; (**D**) composition of each cluster by welding condition.

**Figure 6:**
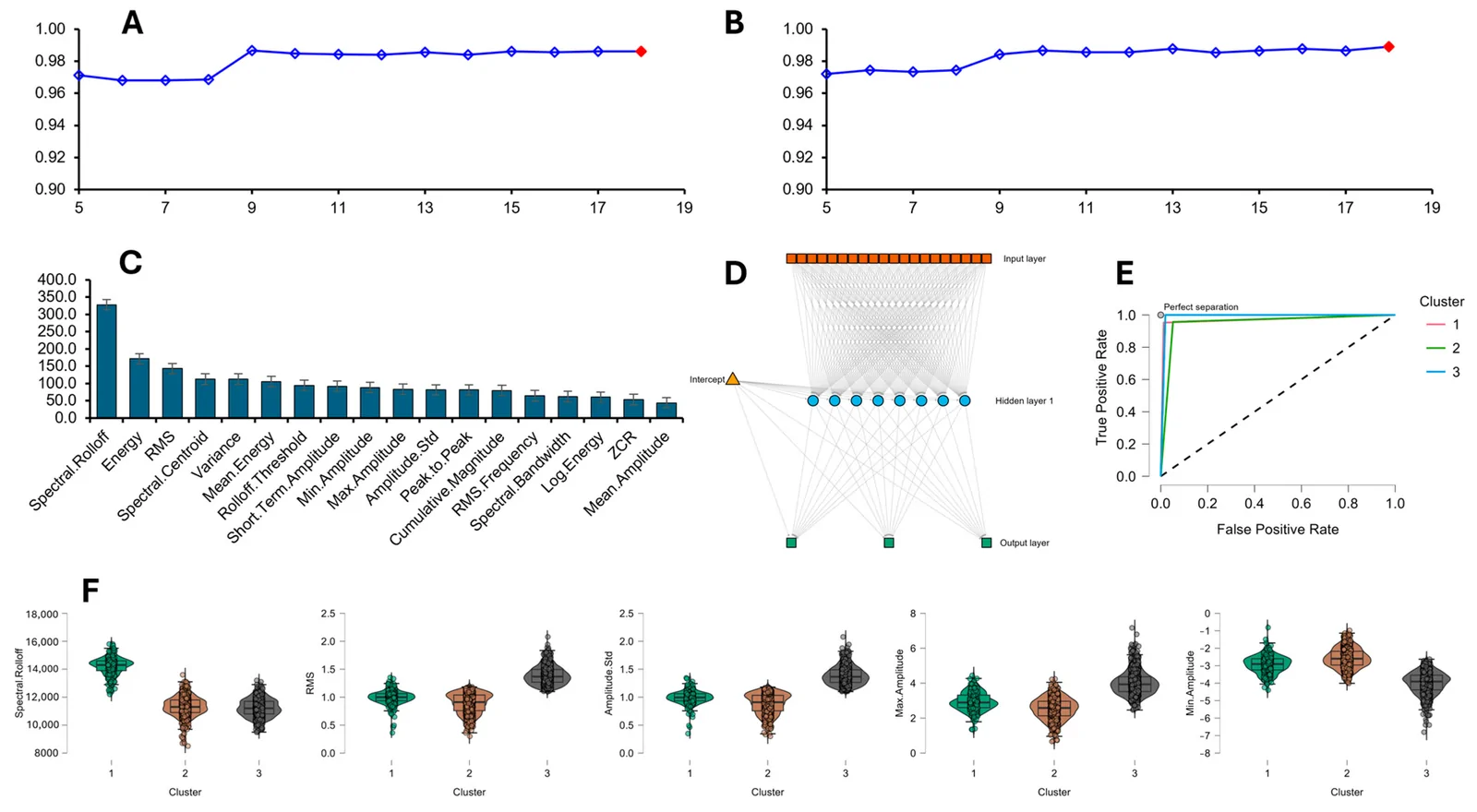
ANN-MLP performance considering (**A**) Validation Accuracy (*y*-axis) and number of features (*x*-axis); (**B**) Test Accuracy (*y*-axis) and number of features (*x*-axis); (**C**) Mean Dropout Loss based on 50 permutations to determine feature importance in MLP-ANN; (**D**) Neural network architecture; (**E**) AUROC curve; (**F**) feature per cluster discrimination.

**Figure 7:**
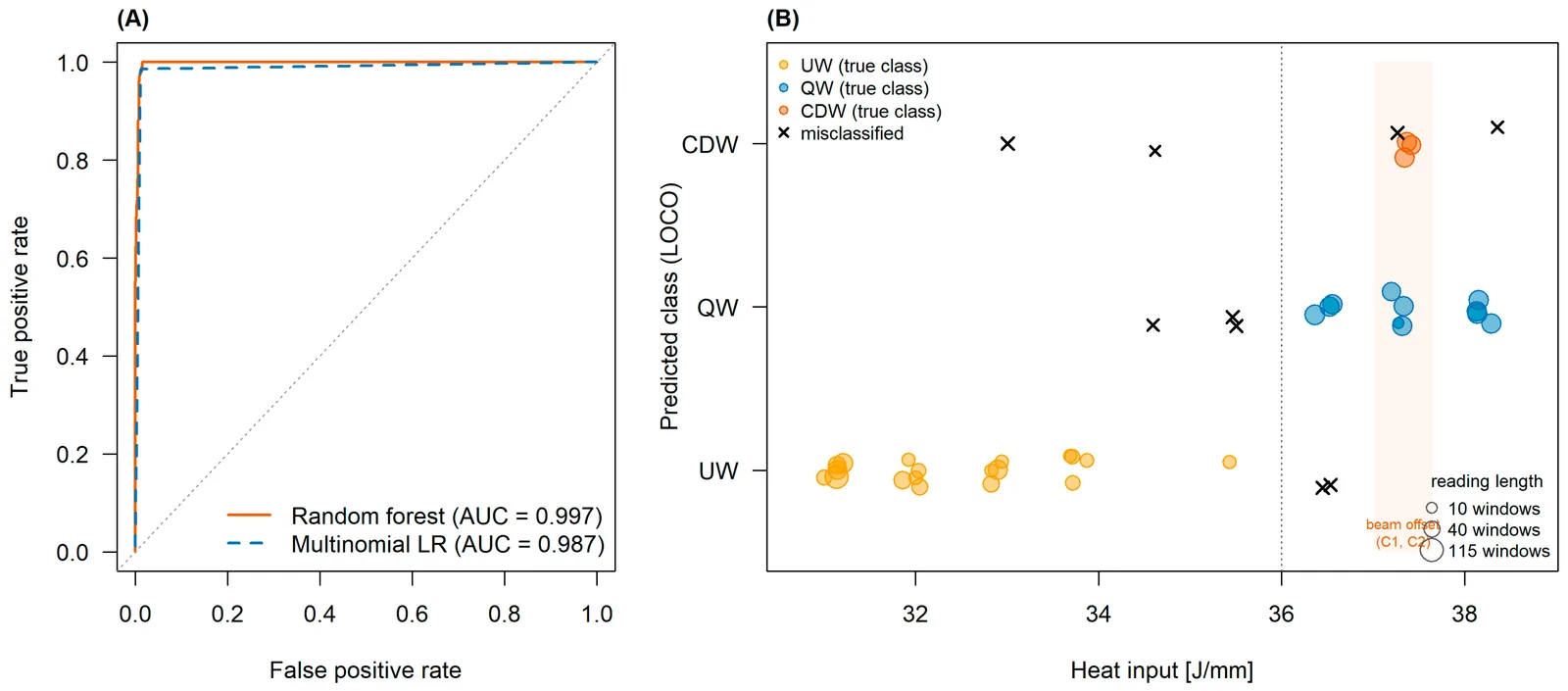
Crack-defective weld detection and weld-level classification under LOCO validation. (**A**) ROC curves for CDW versus non-CDW at window level (RF, MNLR); (**B**) reading-level predicted class against heat input for the aggregated-feature model, with marker size proportional to reading length and misclassified readings marked.

**Figure 8:**
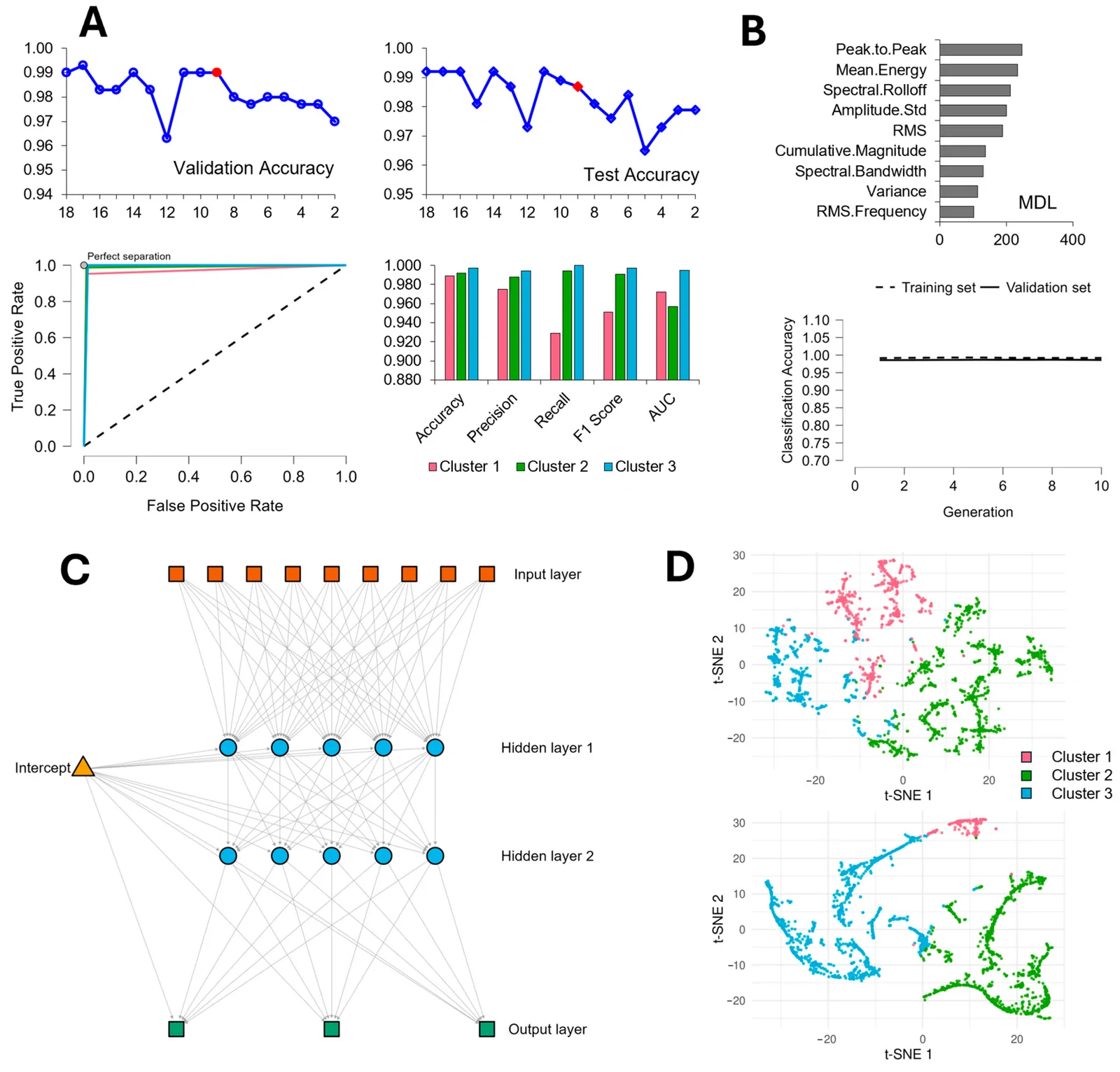
Artificial Neural Network. (**A**) Classification validation and Test Accuracy across clusters (quality categories), including ROC curve and evaluation metrics (accuracy, precision, recall, F1-score, and AUC) for each cluster. (**B**) Feature importance measured by Mean Dropout Loss (**top**) and network classification accuracy plot (**bottom**) after GA optimisation by each generation. (**C**) ANN-MLP network architecture. (**D**) t-SNE visualisation plot of the first hidden layer (**top**) and the second hidden layer (**bottom**) using the first two t-SNE dimensions.

**Figure 9:**
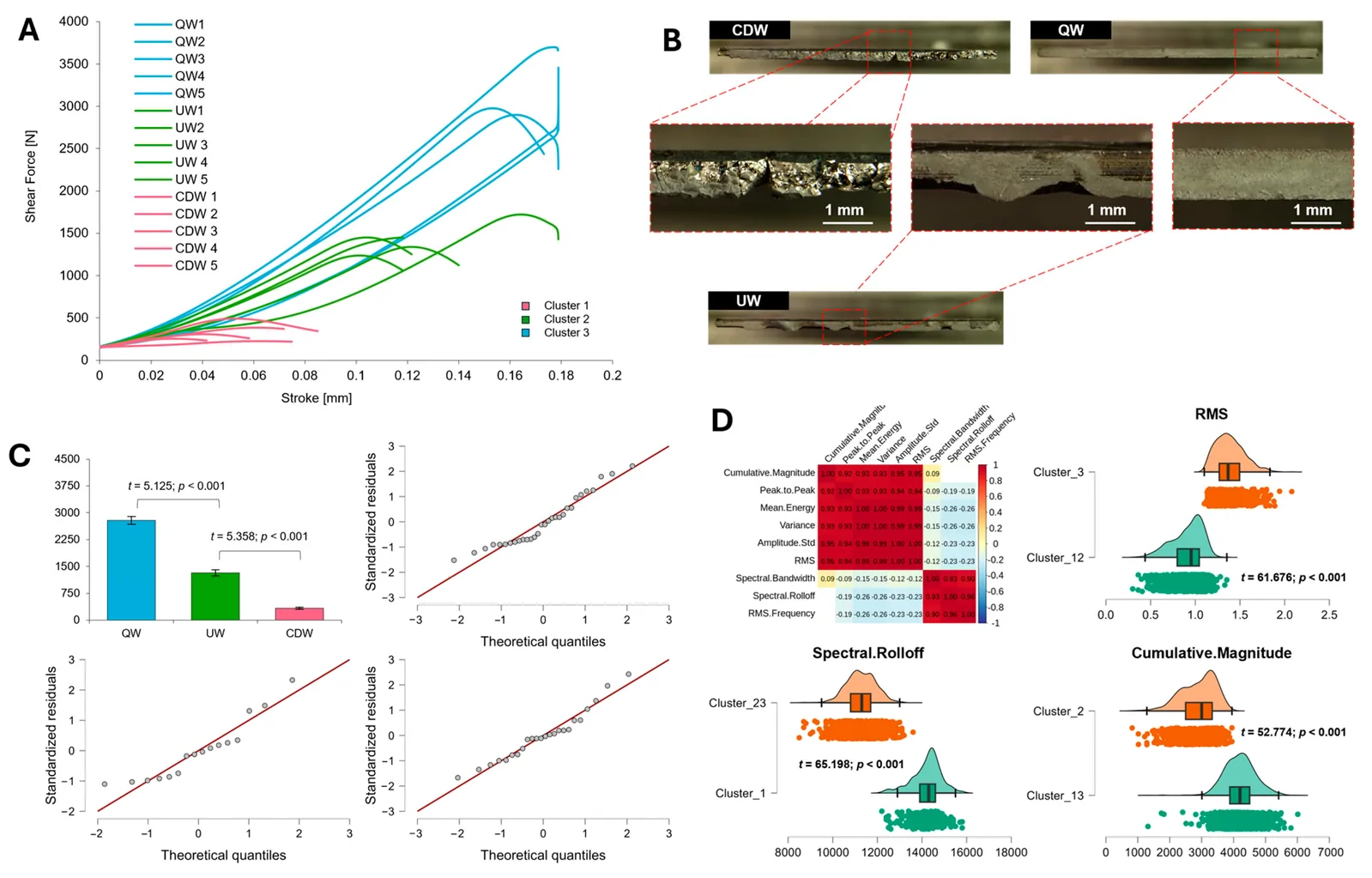
Shear strength validation of weld quality classes. (**A**) Representative load–displacement curves from cluster-derived samples demonstrate a clear separation among the QW, UW, and CDW categories. (**B**) Fracture examination highlights distinct failure morphologies: QW shows a continuous interface with residual cemented carbide on the steel interface, consistent with robust bonding, whereas UW and CDW exhibit incomplete fusion and crack-induced separation. (**C**) Welch’s independent *t*-test confirms statistically significant differences in maximum shear strength between classes, with QW outperforming UW and CDW. (**D**) A correlation heatmap of extracted features and one-vs-rest Welch’s tests identify the most discriminative signal features supporting weld class differentiation.

**Figure 10:**
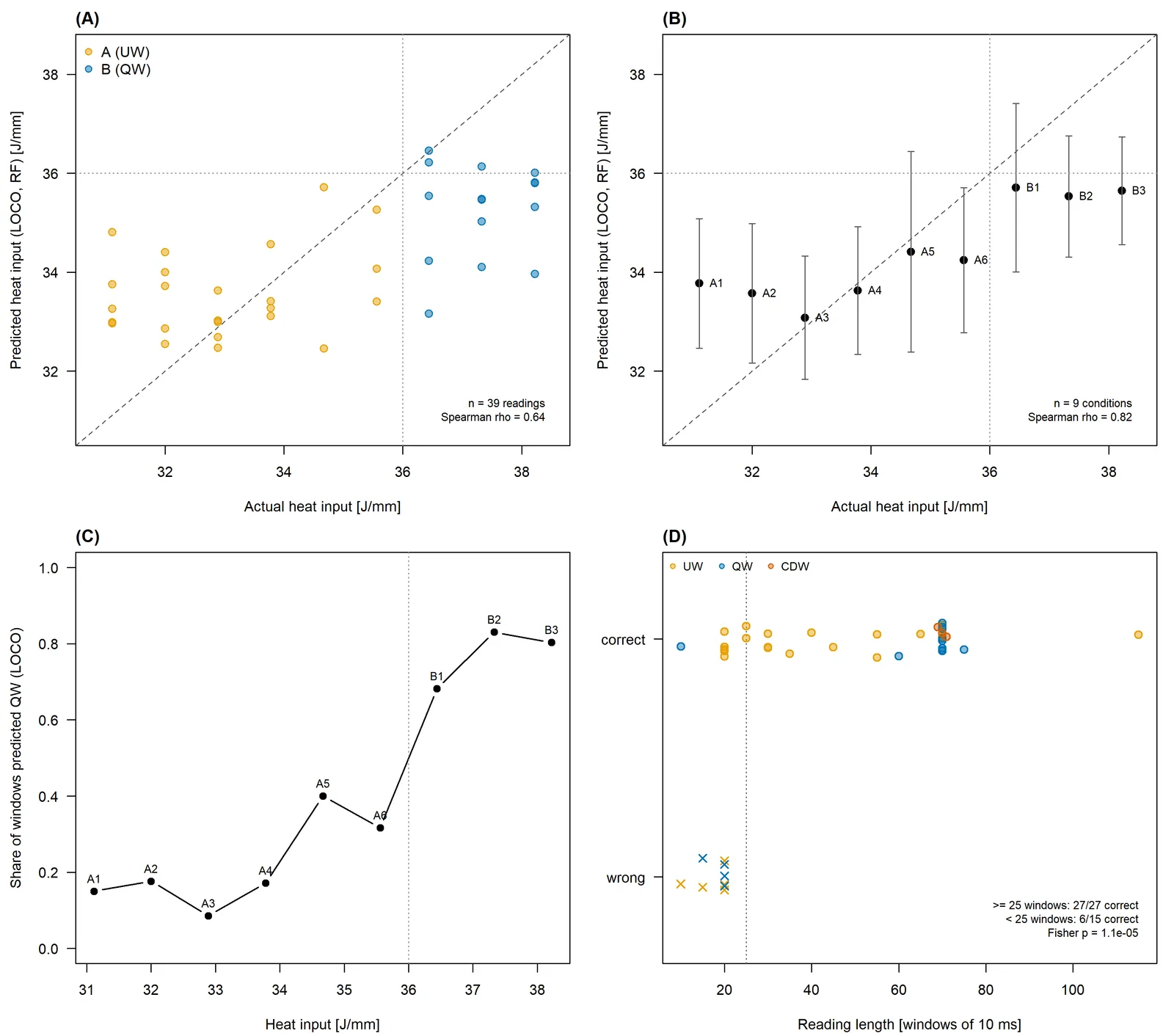
Heat input and observation length. (**A**) Predicted versus actual heat input at reading level and (**B**) at condition level (error bars: standard deviation of window-level predictions) under leave-one-condition-out Random Forest regression; (**C**) share of windows predicted as continuously bonded per condition; (**D**) reading-level classification outcome against reading length. Dotted lines mark the 36 J/mm metallographic threshold and the 25-window (0.25 s) length.

**Table 1:** Literature review summary on AE sensor data monitoring.

Application	Data Analysis	Sensor Data	Outcomes	Ref.
Copper (Cu-ETP and CuSN6)	PCA. Autoencoders. LSTM. BiLSTM. Transformers.	AE. X-ray imaging.	Findings suggest high accuracy (>90%) in detecting weld defects using multispectral emission data from X-ray synchrotron imaging combined with DL methods.	[31]
Steel (AISI304) and Aluminium (ENAW5083)	1D CNN (4 layers).	AE. High-speed imaging.	Conventional methods fail to distinguish the welding condition. CNN achieves >95% accuracy in classifying welding speed and distinguishing material condition. AE signals correlated with spatter formation.	[39]
Copper (Battery cells)	Correlation analysis of weld depth and signal characteristics.	Spectral and AE.	Statistically validated basis for inline process monitoring and quality assurance using statistical features and weld defects (e.g., contamination, misalignment, gaps).	[32]
Steel (22MnB5)	SVM.	AE and Optical Spectra.	Applying acoustic and optical signals in welding significantly improves accuracy and enhances consistency and precision by reducing noise variability.	[33]
Aluminium (AA6061)	1D CNN-LSTM.	AE. High-speed imaging.	AE amplitude and spectrum tracked the keyhole oscillation and penetration state. Classification performance: 99% with VMD + CNN-LSTM.	[52]
High-strength low-alloy steel (HSLA 350)	Logistic Regression vs. Adversarial Tagging (AST, sequence-based).	AE.	Combining AE features with weld heat input yielded high prediction accuracy with AST (91.18%). Demonstrates real-time automated weld defect detection.	[37]
Multilayer steel sheets (S45C)	NA	AE. High-speed imaging.	Establishes that AE signals during welding originate from distinct mechanisms (e.g., weld oscillations), showing that contact conditions influence AE signal propagation.	[34]
Copper- Aluminium	ANN (9 layers).	AE.	Weld strength depends strongly on laser power. Low AE (weak weld), high AE (excessive melt, cracks). ANN yielded 91% accuracy in classifying welds. Demonstrated real-time weld quality prediction of Cu-Al joints.	[44]
Aluminium alloys	kNN. SVM. ANNs. ResNet. VGG-16. Inception v3.	AE.	Predicting weld penetration depth using ResNet, providing interpretable feature localisation through class maps.	[41]
Aluminium (AA5083)	Decision Trees. Ensembles. SVM. ANN.	AE.	Reliable three-class discrimination without requiring complex sensors. Windowed acoustic data (5 s samples) are sufficient for robust classification.	[43]

**Table 2:** Material characteristics [20].

Material	Thermal Expansion [10^−6^/K]	Thermal Conductivity [W/m.K]	Modulus of Elasticity [GPa]
Cr-Mn steel (0.4 wt.% C)	12	45	200
WC-3 wt.% Co	4.6	95	665

**Table 3:** Laser welding parameters.

Case	Heat Input [J/mm]	Laser Beam Position
A1	31.11	Aligned with the steel edge border
A2	32.00	
A3	32.89	
A4	33.78	
A5	34.67	
A6	35.56	
B1	36.44	Aligned with the steel edge border
B2	37.33	
B3	38.22	
C1	37.33	Offset +0.25 mm toward the cemented carbide strip
C2	37.33	Offset +0.5 mm toward the cemented carbide strip

**Table 4:** Time-domain features.

Feature Description	Acronym	Analytical Description (Equation) of the Feature
Root Mean Square	*RMS*	$RMS=\sqrt{\frac{1}{N}\sum_{i=1}^{N}x_{i}^{2}}$
Variance	*Var*	$\sigma^{2}=\frac{1}{N}\sum_{i=1}^{N}{\left(x_{i}-\bar{x}\right)}^{2}$
Amplitude standard deviation	*Amplitude.Std*	$\sigma =\sqrt{\frac{1}{N}\sum_{i=1}^{N}{\left(x_{i}-\bar{x}\right)}^{2}}$
Signal Energy	*Energy*	$E=\sum_{i=1}^{N}x_{i}^{2}$
Mean Energy	*Mean_En*	$\bar{E}=\frac{1}{N}\sum_{i=1}^{N}x_{i}^{2}$
Zero-Crossing Rate	*ZCR*	$ZCR=\frac{1}{N-1}\sum_{i=1}^{N-1}I\left[\left(x_{i}\cdot x_{i+1}\right)<0\right]$, where I[·] is the indicator function: $I\left[P\right]=\left\{\begin{array}{c} 1,\;if\;P\;is\;true\\ 0,\;otherwise \end{array}\right.$
Short-Term Amplitude	*STA*	${STA}_{k}=\frac{1}{M}\sum_{i=k}^{k+M-1}\left|x_{i}\right|,\;for\;k=1,2,\ldots N-M+1$
Peak-to-Peak Amplitude	*P2P*	$P2P=\max_{i}\left(x_{i}\right)-\min_{i}\left(x_{i}\right)$
Mean Amplitude	*Mean*	$Mean=\bar{x}=\frac{1}{N}\sum_{i=1}^{N}x_{i}$
Maximum Amplitude	*Max*	$Max=\max_{i}\left(x_{i}\right)$
Minimum Amplitude	*Min*	$Min=\min_{i}\left(x_{i}\right)$

**Table 5:** Frequency-domain features.

Feature Description	Acronym	Analytical Description (Equation) of the Feature
Spectral Centroid	*Spectral.Centroid*	$f_{cent}=\frac{\sum_{k}f_{k}\omega_{k}}{\sum_{k}\omega_{k}}$
Spectral Bandwidth	*Spectral.Bandwidth*	$BW=\sqrt{\frac{\sum_{k}{\left(f_{k}-f_{cent}\right)}^{2}\omega_{k}}{\sum_{k}\omega_{k}}}$
Spectral Roll-off (at threshold α = 0.85)	*Spectral.Rolloff*	$f_{roll}=min\{f_{m}:E(m)\geq \alpha \sum_{k}\omega_{k}$ roll-off threshold = $\alpha \sum_{k}\omega_{k}$
Cumulative Magnitude	*Cumulative.Mag*	$C[m]=\sum_{k=1}^{m}\left|X\left[k\right]\right|$
RMS Frequency	*Frequency.RMS*	$f_{RMS}=\sqrt{\frac{\sum_{k}{f_{k}}^{2}\omega_{k}}{\sum_{k}\omega_{k}}}$
Log Energy	*Log.Energy*	$E_{log}=log(\sum_{k}\omega_{k}+\varepsilon )$ with small ε > 0

**Table 6:** Model summary: Random Forest classification.

Trees	Features per Split	*n* (Train)	*n* (Validation)	*n* (Test)	Validation Accuracy	Test Accuracy	OOB Accuracy
50	4	1193	299	373	0.987	0.981	0.981

**Table 7:** Model summary: Logistic Regression classification.

Family	Link	*n* (Train)	*n* (Validation)	*n* (Test)	Validation Accuracy	Test Accuracy
Multinomial	Logit function	1193	299	373	0.987	0.981

**Table 8:** Model summary: Support Vector Machine classification.

Violation Cost	Support Vectors	*n* (Train)	*n* (Validation)	*n* (Test)	Validation Accuracy	Test Accuracy
1.880	54	1193	299	373	1.000	0.984

**Table 9:** Model summary: ANN-MLP classification.

Hidden Layers	Nodes	*n* (Train)	*n* (Validation)	*n* (Test)	Validation Accuracy	Test Accuracy
1	8	1193	299	373	0.987	0.987

**Table 10:** Weld-quality prediction under grouped cross-validation.

Task	Scheme	Decision	Model	Accuracy	Bal._acc._	AUC_macro_
CDW vs. non-CDW	LOCO (11)	reading	RF/SVM/MNLR	1.000 (42/42)	1.000	-
CDW vs. non-CDW	LORO (42)	reading	RF/SVM/MNLR	1.000 (42/42)	1.000	-
CDW vs. non-CDW	LOCO (11)	window	MNLR	0.988	0.985	0.987
CDW vs. non-CDW	LORO (42)	window	RF	0.994	0.997	1.000
UW vs. QW (on-axis)	LOCO (9)	reading	RF/SVM/MNLR	0.846 (33/39)	0.825	-
UW vs. QW (on-axis)	LOCO (9)	window	MNLR	0.837	0.836	0.913
UW vs. QW (on-axis)	LORO (39)	window	MNLR	0.845	0.845	0.921
Three-class, two-stage	LOCO (11)	reading	RF/SVM/MNLR	0.857 (36/42)	0.883	-
Three-class, two-stage	LOCO (11)	window	MNLR	0.844	0.878	0.943
Three-class, two-stage	LORO (42)	window	MNLR	0.859	0.893	0.954
Three-class, aggregated	LOCO (11)	reading	RF	0.786	0.842	0.942
Three-class, aggregated	LORO (42)	reading	SVM	0.833	0.869	0.925
Reference: cluster labels	random 60/20/20	window	ANN-MLP	0.987	-	1.000

**Table 11:** Reading-level errors by welding condition (aggregated-feature model, LOCO).

Condition	Heat Input [J/mm]	Class	Readings	Windows per Reading	Errors
A1	31.11	UW	5	35–115	0
A2	32.00	UW	5	20–55	0
A3	32.89	UW	5	20–70	1 (20 windows)
A4	33.78	UW	4	20–30	0
A5	34.67	UW	2	10–15	2 (10, 15)
A6	35.56	UW	3	20	2 (20, 20)
B1	36.44	QW	5	20–75	2 (20, 20)
B2	37.33	QW	5	10–70	1 (20)
B3	38.22	QW	5	15–70	1 (15)
C1	37.33 (+0.25 mm)	CDW	1	69	0
C2	37.33 (+0.50 mm)	CDW	2	70–71	0
Total			42	10–115	9
Condition	Heat input [J/mm]	Class	Readings	Windows per reading	Errors

**Figure A1:**
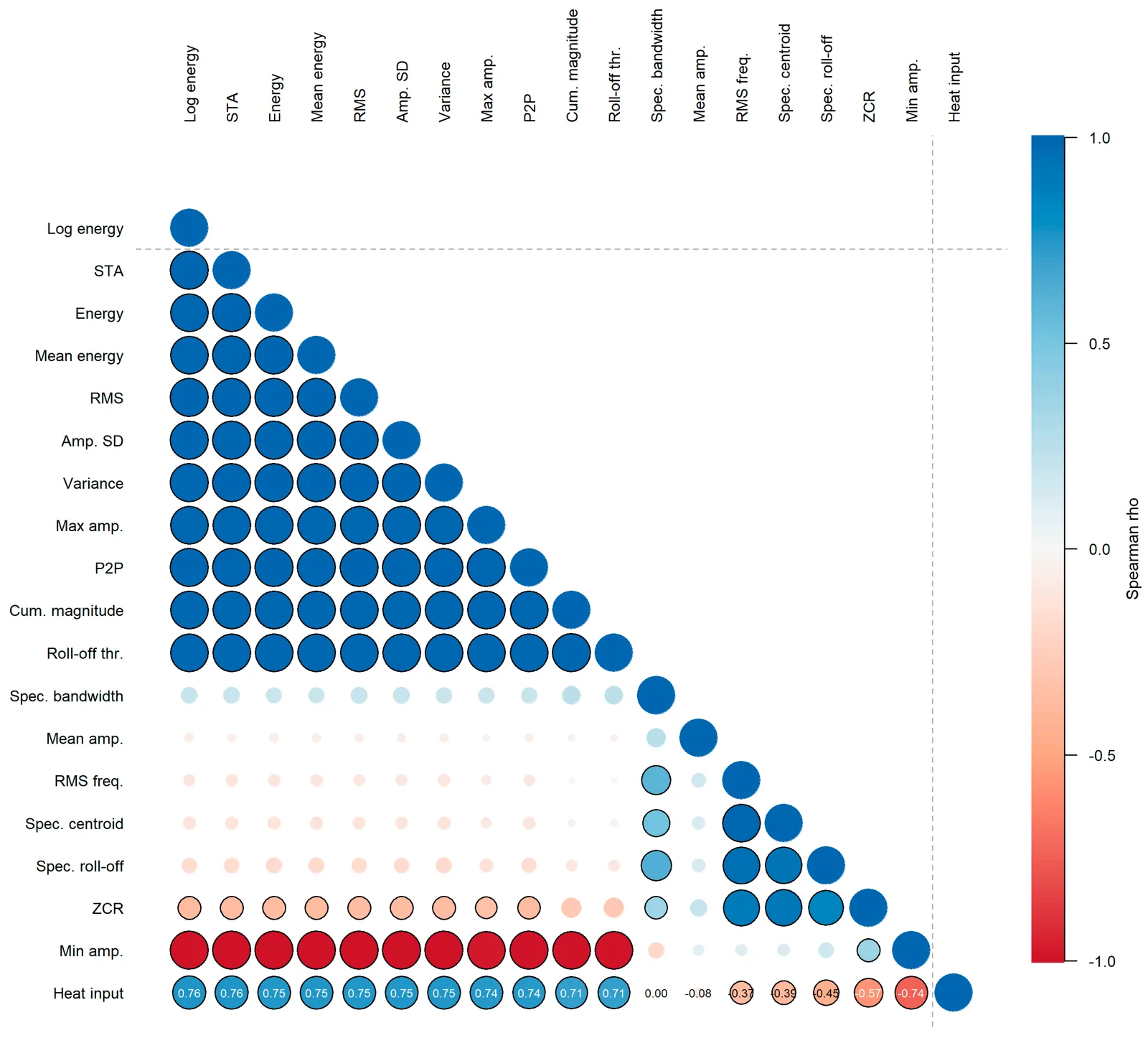
Spearman correlation heatmap among 18 reading-mean features and heat input.

## Data Availability

The data presented in this study are available on request from the corresponding author due to the fact that they are part of ongoing research.

## Abbreviations

The following abbreviations are used in this manuscript:

Acronym	Description
AE	Acoustic Emission
AHC	Agglomerative Hierarchical Clustering
ANN	Artificial Neural Network
AUC	Area Under the Receiver Operating Characteristic Curve
BP	Backpropagation
BPANN	Backpropagation Artificial Neural Network
CDW	Crack-Defective Weld
CNN	Convolutional Neural Network
FFT	Fast Fourier Transform
GA	Genetic Algorithm
HAZ	Heat Affected Zone
HCPC	Hierarchical Clustering on Principal Components
HM	Hard Metal
LDA	Linear Discriminant Analysis
LOCO	Leave-One-Condition-Out
LORO	Leave-One-Reading-Out
LR	Logistic Regression
LSTM	Long-Short Term Memory
MDL	Mean Dropout Loss
ML	Machine Learning
MLP	Multi-Layer Perceptron
MNLR	Multinomial Logistic Regression
OOB	Out-of-Bag
PCA	Principal Component Analysis
QW	Quality Weld
RFE	Recursive Feature Elimination
RF	Random Forest
ROC	Receiver Operating Characteristic
ROI	Region of Interest
RMS	Root Mean Square
SE	Standard Error
SVM	Support Vector Machine
TEDS	Transducer Electronic Data Sheet
UW	Unstable Weld
WZ	Weld Zone

## Appendix A

**Table A1:** Composition of the three HCPC clusters by welding condition (column percentage of 10 ms windows). The CDW cluster contains every window from C1 and C2. Purity 0.846; adjusted Rand index 0.543.

Cluster	A1	A2	A3	A4	A5	A6	B1	B2	B3	C1	C2
UW	81	80	89	78	48	58	19	12	6	0	0
QW	16	19	10	20	48	35	78	85	94	0	0
CDW	2	1	1	2	4	7	3	3	0	100	100

## Appendix B

**Table A2:** ANN-MLP hyperparameter settings with manual settings of network topology.

Function	Algorithm	Learning Rate	Layers	Nodes	Acc_test_	Acc_valid._	Precision	Recall	F1score	AUC
ReLU	Rprop+	-	1	9	0.984	0.989	0.985	0.94	0.984	0.983
tanh	Rprop+	-	1	9	0.987	0.991	0.987	0.987	0.986	0.988
GeLU	Rprop+	-	1	9	0.408	0.605	0.454	0.408	0.402	0.531
ReLU	Rprop+	-	2	10;5	0.987	0.991	0.987	0.987	0.987	0.706
tanh	Rprop+	-	2	10;5	0.984	0.989	0.984	0.984	0.984	0.984
tanh	Rprop−	-	2	10;5	0.987	0.991	0.987	0.987	0.987	0.983
Softplus	Rprop+	-	1	7	0.979	0.986	0.979	0.979	0.979	0.989
Mish	Rprop+	-	1	7	0.705	0.803	0.815	0.705	0.687	0.749
ReLU	BP	0.01	1	9	0.335	0.557	0.420	0.335	0.359	0.501
ReLU	BP	0.005	1	9	0.335	0.557	0.420	0.335	0.359	0.501
ReLU	BP	0.01	2	10;5	0.332	0.555	0.411	0.332	0.356	0.492
tanh	BP	0.01	2	10;5	0.378	0.585	0.383	0.378	0.373	0.621
log-sigmoid	BP	0.01	1	9	0.984	0.989	0.984	0.984	0.984	0.982
log-sigmoid	BP	0.01	2	10;5	0.992	0.995	0.992	0.992	0.992	0.985
log-sigmoid	Rprop+	-	2	10;5	0.992	0.995	0.992	0.992	0.992	0.987
log-sigmoid	Rprop−	-	2	10;5	0.989	0.993	0.989	0.989	0.989	0.986
log-sigmoid	Gprop-slr	-	2	10;5	0.992	0.995	0.992	0.992	0.992	0.988
log-sigmoid	Gprop-sag	-	2	10;5	0.992	0.995	0.992	0.992	0.992	0.990
tanh	Gprop-sag	-	2	10;5	0.992	0.995	0.992	0.992	0.992	0.978
tanh	Gprop-sag	-	1	9	0.987	0.991	0.987	0.987	0.986	0.987
tanh	Gprop-slr	-	1	9	0.979	0.986	0.979	0.979	0.978	0.987
tanh	Gprop-slr	-	2	10;5	0.992	0.995	0.992	0.992	0.992	0.992

**Table A3:** Optimisation of network topology with different Genetic Algorithm settings.

Algorithm	Nd	Parent Selection	Candidates	Crossover	Mutation	Survival	Acc_Test_	Acc_Valid._	Precision	Recall	F1score	AUC
Rprop+	8;2	Roulette wheel	-	Uniform	Reset	Fitness-based	0.979	0.997	0.979	0.979	0.979	0.986
Rprop−	4	Roulette wheel	-	Uniform	Reset	Fitness-based	0.989	0.993	0.989	0.989	0.989	0.987
Gprop-sag	4;9	Roulette wheel	-	Uniform	Reset	Fitness-based	0.987	0.993	0.991	0.987	0.987	0.987
Gporp-slr	4	Roulette wheel	-	Uniform	Reset	Fitness-based	0.987	0.983	0.991	0.987	0.987	0.980
Rprop−	7	Universal	-	Uniform	Reset	Fitness-based	0.989	0.993	0.989	0.989	0.989	0.985
Gprop-slr	9;7	Universal	-	Uniform	Reset	Fitness-based	0.984	0.987	0.984	0.984	0.984	0.984
Gprop-sag	6	Universal	-	Uniform	Reset	Fitness-based	0.989	0.993	0.989	0.989	0.989	0.980
Rprop+	6	Tournament	5	Uniform	Reset	Fitness-based	0.987	0.990	0.987	0.987	0.987	0.984
Rprop+	4;9	Tournament	5	One-point	Reset	Fitness-based	0.989	0.990	0.989	0.989	0.989	0.988
Rprop−	8;8	Tournament	5	One-point	Reset	Fitness-based	0.989	0.993	0.989	0.989	0.989	0.986
Gprop-sag	9;4	Tournament	5	One-point	Reset	Fitness-based	0.984	0.993	0.984	0.984	0.984	0.984
^A^ Rprop+	4;9	Tournament	5	One-point	Swap	Fitness-based	0.992	0.990	0.992	0.992	0.992	0.981
^B^ Rprop+	9;10	Tournament	5	One-point	Swap	Fitness-based	0.987	0.973	0.987	0.987	0.987	0.979
Rprop+	10;9	Tournament	5	One-point	Swap	Random	0.984	0.991	0.987	0.987	0.987	0.987
Gprop-sag	10;9	Tournament	5	One-point	Swap	Random	0.989	0.990	0.989	0.989	0.989	0.981
Rprop−	4;6	Tournament	5	One-point	Swap	Random	0.984	0.993	0.984	0.984	0.984	0.984
^A^ Gprop-slr	9;4	Tournament	5	One-point	Swap	Age-based	0.992	0.990	0.992	0.992	0.992	0.985
^B^ Gprop-slr	9;4	Tournament	5	One-point	Swap	Age-based	0.987	0.983	0.987	0.987	0.987	0.975
Rprop+	4	Tournament	5	Uniform	Swap	Age-based	0.987	0.993	0.987	0.987	0.987	0.985
Rprop+	9;9	Tournament	5	One-point	Swap	Age-based	0.989	0.993	0.989	0.989	0.989	0.985
^A^ Gprop-slr	10;4	Tournament	5	One-point	Swap	Age-based	0.992	0.990	0.992	0.992	0.992	0.985
^B^ Gprop-slr	7;7	Tournament	5	One-point	Swap	Age-based	0.987	0.983	0.987	0.987	0.987	0.975
Gprop-sag	4;4	Tournament	5	One-point	Reset	Age-based	0.989	1.000	0.989	0.989	0.989	0.982
^A^ Gprop-slr	10;4	Tournament	5	One-point	Swap	Age-based	0.992	0.990	0.992	0.992	0.992	0.985
^B^ Gprop-slr	10;4	Tournament	5	One-point	Swap	Age-based	0.987	0.983	0.987	0.987	0.987	0.975
^A^ Gprop-slr	3;3	Universal	-	One-point	Swap	Age-based	0.992	0.997	0.992	0.992	0.992	0.985
^B^ Gprop-slr	8;8	Universal	-	One-point	Swap	Age-based	0.984	0.987	0.984	0.984	0.984	0.978

Note: Nd = Nodes in layers 1 and 2, respectively. Acc = Accuracy of the test and validation dataset. AUC = Area Under the Receiver Operating Characteristic Curve. ^A,B^ Explains the seed set at random numbers A = 1234 and B = 2801.
